# Supplementary material for: Efficient spatio-angular reconstruction enables high-fidelity mapping of six-dimensional structures and dynamics with polarized fluorescence microscopy
Source: Nat Commun. 2026 Aug 17;17:9853. doi: 10.1038/s41467-026-76703-0 (PMC13578705; doi:10.1038/s41467-026-76703-0)
Supplement: Supplementary file 2 [file 41467_2026_76703_MOESM2_ESM.pdf]

Description of Additional Supplementary Files

Supplementary Video 1, Rendering methods for clearer visualization of spatio-angular objects. First, we synthesize a spatio-angular shell phantom consisting of dumbbell shaped ODFs oriented along the radial direction at each surface voxel. Then, volumetric (left column), sliced (middle column) and fly-around (right column) ODF/peak orientation maps are rendered under different thresholds and down-sampling ratios, where the orientations in the peak orientation maps are represented by both the directions and the pseudo colors of cylinders. See also **Supplementary Fig. 1a**.

Supplementary Video 2, Demonstration of spatio-angular reconstruction process on synthetic phantom with eGRL. Two parallel lines in 3D space with two different kinds of ODFs in each line (1st column, dumbbell-shaped in top line and x-shaped in bottom line) are imaged with polarized diSPIM. Here only a transverse XY plane through the object is shown under one specific polarization modulation. The spatial and angular distribution are jointly reconstructed by eGRL, ODFs in the two lines (2nd column) and line profiles through the middle of the image (3rd and 4th column) are also shown and updated after each iteration count. See also **Fig. 1b**.

Supplementary Video 3, Performance of eGRL vs. SVD on synthetic data with different noise levels. A synthetic shell phantom is imaged with polarized dual-view light sheet microscopy and the raw data are corrupted with different levels of Poisson noise, then separately reconstructed by eGRL (10 iterations) and SVD (0.01 regularization parameter). Note eGRL reconstruction is robust to noise while SVD is quite sensitive to noise. See also **Supplementary Fig. 9**.

Supplementary Video 4, Bias errors caused by regularization parameter in SVD reconstruction on simulated data. Synthetic shell phantom with molecular orientations perpendicular to the surface (left). SVD reconstructions at different regularization parameters η ('eta') values are visualized in the peak orientations (middle) and the radar chart (right) counts the x, y and z proportion of all peak orientations. Increasing the regularization parameter results in structural distortion and orientations biased towards the y-axis. See also **Fig. 2g**.

Supplementary Video 5, Experimental GUV labelled with FM1-43, showing raw data and reconstruction process. Raw images with 18 polarization modulations (9 volumes for each view) were acquired with pol-diSPIM (left column), highlighting local differences in intensity in different subregions of the same structure. Taking the average of all raw data as the initial

estimate, eGRL iterations jointly estimate the peak orientation (middle column) and density (right column) distributions. All the density images are shown from the same middle slice (z = 10.5 µm) selected from the 3D volume. See also **Fig. 3a**.

Supplementary Video 6, Characterizing orientational preference of FM1-43 anchored to GUV membrane. As in **Supplementary Video 5**, but shown in slices. Each layer along the y-axis is visualized by slicing (left column), showing the ODF (middle column) and peak (right column) orientations aligned perpendicular to the shell. See also **Fig. 3a**, **b**.

Supplementary Video 7, Fly-around demonstration of orientation distribution in GUV samples. As in **Supplementary Video 5**, but showing the reconstructions of the whole GUV dataset (1st column) by eGRL vs. SVD estimation, and 3 representative ROIs (2nd – 4th column) for higher magnification observation. A color-coded sphere is visualized at the corner and synchronously rotated to indicate which orientation a specific color represents. See also **Fig. 3a**, **b**.

Supplementary Video 8, eGRL provides better structural contrast and more accurate orientation distribution than SVD when applied to fixed U2OS samples labelled with Alexa Fluor 488 phalloidin. eGRL and SVD results are shown in terms of density (left column), ODF (middle column) and peak orientation (right column) maps. See also **Fig. 3c-e**.

Supplementary Video 9, eGRL still performs well with reduced polarization measurements on tobacco xylem cells labelled with Pontamine fast scarlet. Raw images were captured with 18, 8 and 6 polarization modulations and separately reconstructed by both eGRL and SVD. Note the downsampling in polarization encoding shows that eGRL is robust to the accurate orientation details. See also **Fig. 3i.**

Supplementary Video 10, eGRL performs well with reduced polarization measurements on fixed U2OS labelled with Alexa Fluor 488 phalloidin. Raw images were captured on fixed U2OS cells under 18, 8 and 6 polarization modulations and separately reconstructed by both eGRL (top) and SVD (bottom). See also **Supplementary Fig. 14**.

Supplementary Video 11, Visualizing Xylem cells labeled with Pontamine fast scarlet. Orientation map of Xylem cell was reconstructed with eGRL and SVD. We also show slices taken along the y axis of the ODF and orientation map. See also **Fig. 3h**.

Supplementary Video 12, eGRL retrieves angular rotational-distribution and dynamics of SiR Actin in live HeLa cells, that are obscured in intensity images. Density map (left) and maximum-intensity-projection pseudo-colored by orientation (middle). The polar charts (right) indicate the orientation distribution along the trails on peripheral (yellow border) and protrusion (green border) regions respectively represented by 50 and 25 selected points in the density map. Low fluctuations along peripheral region serve as a control to assess orientation dynamics caused by non-cellular changes, e.g. photobleaching. Dynamics along protrusion demonstrate small changes that are obscured in the intensity images, but noticeable in the orientation distribution. See also **Fig. 5a-c**.

Supplementary Video 13, Membrane transitions, as assessed via morphology and orientation, in a single live macrophage cell labeled with FM1-43. Time-lapse maximum intensity projection of density (left) and orientation distribution (middle), revealing the transition from membrane protrusion to expanding bleb (magnified in right column). See also **Supplementary Fig. 20**.

Supplementary Video 14, eGRL restores spatio-angular dynamics on membrane protrusions in living macrophages. First, fly-around density and orientation maps at initial time point to characterize the orientational alignment of FM1-43 probe on live macrophage samples. Then, z-slices of dual-view raw data from pol-diSPIM are compared with estimated and orientation distribution. Last, we show time-lapse maximum intensity projections of reconstructed density maps that are pseudo-colored by orientation. See also **Fig. 5d-e.**

Supplementary Video 15, Dual-color spatio-angular imaging of live U2OS cells labeled by FM1-43 and RFP-PH dyes. Time-lapse maximum intensity projection of dual-color raw data (1st column), reconstructed orientation (2nd and 3rd columns) and GFA (4th and 5th columns) maps in FM1-43 and RFP-PH channels. Note GFA extracted from spatio-angular reconstructions by eGRL provides a quantitative measure of molecular distribution anisotropy beyond principal-orientation representations. See also **Supplementary Fig. 22.**

Supplementary Video 16, eGRL reconstructs spatio-angular dynamics on live U2OS cell membrane labeled by FM1-43. First, successive image planes of pol-diSPIM raw data and eGRL reconstructed spatio-angular distribution at time point 0. Then, rendering of time-lapse volumetric density and orientation map, showing a higher magnification view of the cell substrate to highlight the orientational alignment that accompanies the membrane expansion. See also **Fig. 5f-k.**
