# Supplementary material for: Efficient spatio-angular reconstruction enables high-fidelity mapping of six-dimensional structures and dynamics with polarized fluorescence microscopy
Source: Nat Commun. 2026 Aug 17;17:9853. doi: 10.1038/s41467-026-76703-0 (PMC13578705; doi:10.1038/s41467-026-76703-0)
Supplement: Supplementary file 3 [file 41467_2026_76703_MOESM4_ESM.pdf]

nature portfolio

Corresponding author(s): Huafeng Liu and Min Guo

Last updated by author(s): Jul 20, 2026

Reporting Summary

Nature Portfolio wishes to improve the reproducibility of the work that we publish. This form provides structure for consistency and transparency in reporting. For further information on Nature Portfolio policies, see our Editorial Policies and the Editorial Policy Checklist.

Statistics

For all statistical analyses, confirm that the following items are present in the figure legend, table legend, main text, or Methods section.

n/a	Confirmed	
☐	☒	The exact sample size (*n*) for each experimental group/condition, given as a discrete number and unit of measurement
☐	☒	A statement on whether measurements were taken from distinct samples or whether the same sample was measured repeatedly
☒	☐	The statistical test(s) used AND whether they are one- or two-sided<br>*Only common tests should be described solely by name; describe more complex techniques in the Methods section.*
☒	☐	A description of all covariates tested
☒	☐	A description of any assumptions or corrections, such as tests of normality and adjustment for multiple comparisons
☐	☒	A full description of the statistical parameters including central tendency (e.g. means) or other basic estimates (e.g. regression coefficient) AND variation (e.g. standard deviation) or associated estimates of uncertainty (e.g. confidence intervals)
☒	☐	For null hypothesis testing, the test statistic (e.g. *F*, *t*, *r*) with confidence intervals, effect sizes, degrees of freedom and *P* value noted<br>*Give P values as exact values whenever suitable.*
☒	☐	For Bayesian analysis, information on the choice of priors and Markov chain Monte Carlo settings
☒	☐	For hierarchical and complex designs, identification of the appropriate level for tests and full reporting of outcomes
☒	☐	Estimates of effect sizes (e.g. Cohen's *d*, Pearson's *r*), indicating how they were calculated

Our web collection on statistics for biologists contains articles on many of the points above.

Software and code

Policy information about availability of computer code

Data collection	The simulated data of phantom objects and spatio-angular imaging results were generated with Python 3.9 and the code is publicly available at https://github.com/ejunyuliu/PFM-tool.<br>Experimental images were collected with the home-built polarized light sheet microscope (pol-diSPIM) as described in the Methods section of our paper.
Data analysis	Generalized Richarson Lucy algorithm (GRL) and efficient generalized Richarson Lucy algorithm (eGRL) are publicly available at https://github.com/ejunyuliu/PFM-tool. Singular Value Decomposition algorithm (SVD) was installed from Github (https://github.com/talonchandler/polaris).<br>Other analysis code (e.g., calculation of SNR, SSIM, FWHM, PSIM, OSIM) was written in Python 3.9, and is available upon request.

For manuscripts utilizing custom algorithms or software that are central to the research but not yet described in published literature, software must be made available to editors and reviewers. We strongly encourage code deposition in a community repository (e.g. GitHub). See the Nature Portfolio guidelines for submitting code & software for further information.

Data

Policy information about availability of data

All manuscripts must include a data availability statement. This statement should provide the following information, where applicable:

- Accession codes, unique identifiers, or web links for publicly available datasets
- A description of any restrictions on data availability
- For clinical datasets or third party data, please ensure that the statement adheres to our policy

The data that support the findings of this study are included in Figs. 1-5, Supplementary Figs. 1–26 and Supplementary Videos 1–16. Representative data to test the code are publicly available at https://doi.org/10.5281/zenodo.17033756; datasets of fixed cells are available at https://www.ebi.ac.uk/biostudies/bioimages/studies/S-BIAD1055; other datasets of live cells are available from the corresponding author upon request due to their large size. Source Data are provided with this paper.

Research involving human participants, their data, or biological material

Policy information about studies with human participants or human data. See also policy information about sex, gender (identity/presentation), and sexual orientation and race, ethnicity and racism.

Reporting on sex and gender	N/A
Reporting on race, ethnicity, or other socially relevant groupings	N/A
Population characteristics	N/A
Recruitment	N/A
Ethics oversight	N/A

Note that full information on the approval of the study protocol must also be provided in the manuscript.

Field-specific reporting

Please select the one below that is the best fit for your research. If you are not sure, read the appropriate sections before making your selection.

☒ Life sciences ☐ Behavioural & social sciences ☐ Ecological, evolutionary & environmental sciences

For a reference copy of the document with all sections, see nature.com/documents/nr-reporting-summary-flat.pdf

Life sciences study design

All studies must disclose on these points even when the disclosure is negative.

Sample size

To ensure biological reproducibility, all experiments were performed in at least three independent biological replicates. Because our observations focused on reconstruction algorithm, we analyzed four different cell types to accurately capture population heterogeneity.

Fig. 2f, SSIM, PSIM and OSIM quantifications comparing SVD and eGRL were repeated n=10 times.
Fig. 3i, statistical analysis of the angle between peak orientations and ribs was performed on n=268 (rib1) and n=263 (rib2) points.
Fig. 5c i, scatter plots of angular distributions were generated from n=50 points on protrusions.
Fig. 5c ii, statistical analysis of angular dynamics was performed on n=39, 39, 27, 39 and 38 points for protrusion and actin 1–4, respectively.
Fig. 5h, histograms of GFA values were generated from n=7475 and n=4964 points on membranes near the coverslip and elsewhere, respectively.
Sup. Fig. 3b, FWHM measurements on beads were performed on n=10 individual beads.
Sup. Fig. 4, Monte Carlo simulations were performed on 1620 images with 350 ODFs per image, yielding a total of n=567000 ODFs.
Sup. Fig. 5, quantitative comparisons between eGRL and GRL were repeated n=5 times.
Sup. Fig. 9, performance evaluations of eGRL and SVD were repeated n=5 times.
Sup. Fig. 12, histograms of GFA values were generated from the entire volume, corresponding to a total of n=107008000 voxels.
Sup. Fig. 18, polar plots of angular distributions and dynamics were generated from n=50 characteristic points on protrusions.
Sup. Fig. 22d, averaged GFA values were quantified from n=3 samples, with 30 time points acquired for each sample.
Sup. Fig. 23b, fidelity was quantified by PSIM for mismatches along the x, y and z directions, repeated n=6 times.
Sup. Fig. 24c, fidelity was quantified by PSIM for random mismatches, repeated n=10 times.
Sup. Fig. 25c, convergence of eGRL iterations was evaluated on GUV samples using n=10 randomly selected regions.
Sup. Fig. 26b, the effect of initialization strategies in eGRL iterations was evaluated on randomly distributed spherical phantoms, repeated n=10 times.

Data exclusions

No data were excluded from the analysis.

Replication	The reproducibility of the experimental findings was verified by imaging distinct fixed and live samples with sample size N>=3. Time-lapse imaging experiments were repeated at least 3 times, with similar results obtained each time. All attempts at replication were successful.
Randomization	In this study, samples were not allocated into different experimental groups.
Blinding	The investigators were not blinded to group allocation during data collection and data analysis. We don't think blinding is relevant in this study and we demonstrated the technique on distinct samples by collaborating with different research groups.

Reporting for specific materials, systems and methods

We require information from authors about some types of materials, experimental systems and methods used in many studies. Here, indicate whether each material, system or method listed is relevant to your study. If you are not sure if a list item applies to your research, read the appropriate section before selecting a response.

Materials & experimental systems

n/a	Involved in the study
☒	☐ Antibodies
☐	☒ Eukaryotic cell lines
☒	☐ Palaeontology and archaeology
☒	☐ Animals and other organisms
☒	☐ Clinical data
☒	☐ Dual use research of concern
☒	☐ Plants

Methods

n/a	Involved in the study
☒	☐ ChIP-seq
☒	☐ Flow cytometry
☒	☐ MRI-based neuroimaging

Eukaryotic cell lines

Policy information about cell lines and Sex and Gender in Research

Cell line source(s)	The following cell lines were used in this study: U2OS (ATCC, HTB-96), NIH3T3 (ATCC, CRL-1658), HeLa (ATCC, HTB-96), and macrophage (ATCC, A1049101).
Authentication	None of the cell lines used were authenticated.
Mycoplasma contamination	The cell lines were not tested for mycoplasma contamination.
Commonly misidentified lines (See ICLAC register)	No commonly misidentified cell lines were used in this study.

Plants

Seed stocks	N/A
Novel plant genotypes	N/A
Authentication	N/A
