# Supplementary material for: Efficient spatio-angular reconstruction enables high-fidelity mapping of six-dimensional structures and dynamics with polarized fluorescence microscopy
Source: Nat Commun. 2026 Aug 17;17:9853. doi: 10.1038/s41467-026-76703-0 (PMC13578705; doi:10.1038/s41467-026-76703-0)
Supplement: Supplementary file 4 [file 41467_2026_76703_MOESM5_ESM.pdf]

nature portfolio

Peer Review File

Efficient spatio-angular reconstruction enables high-fidelity mapping of six-dimensional structures and dynamics with polarized fluorescence microscopy

Corresponding Author: Professor Min Guo

This file contains all reviewer reports in order by version, followed by all author rebuttals in order by version.

Version 0:

Reviewer comments:

Reviewer #1

(Remarks to the Author)
See attached.

(Remarks on code availability)

Reviewer #2

(Remarks to the Author)
In this work, the authors develop a method to image three-dimensional orientation properties of fluorophores in lipid membranes and cell samples, using volumetric imaging microscopy. Polarization resolved light sheet imaging, in particular using a polarized dual-view light-sheet system, leads to a full 6D spatio-angular information with reliable reconstruction. The work is mostly concentrated on the improvement of the data processing method as compared to a previously published work by the authors (Proceedings of the National Academy of Sciences, 2025). This method, based on an efficient generalized Richardson-Lucy (eGRL) algorithm, makes a convincing use of spherical harmonics decompositions, exhibits high quality results and a clear advantage over previous approaches in terms of processing speed and efficiency. The theoretical analysis is based on well-chosen mimicking phantom objects, whose shape and orientational distributions can be challenging to measure in 3D. The work is sound and well presented despite a few unclear aspects as detailed below. The robustness of the method and its increased speed opens the way to measurements of structures in 3D in live samples, which is a novelty.

1. The theoretical study of Fig. 1e displays a comparison between the ground truth on helical patterns and their reconstruction. The message given from it and other theoretical figures throughout the work is rather qualitative, it is difficult to see how much the reconstruction deviates from the ground truth. For instance, while the color evolution along the helices seem to be nicely reproduced, some lines are clearly oriented differently and of different lengths than the ground truth : it would be good to have a more quantitative idea of what these differences represent. Monte Carlo simulations could be used to quantify both accuracy and precision on all retrieved parameters, their inter-dependence and their sensitivity to signal to noise levels. Similarly, order parameters values can be very low but their precision is unknown (for ex. in Suppl Fig S15-iii). Graphs showing accuracy and precision values for both orientation and anisotropy (GFA or OP) for different kinds of phantoms displayed in Fig. 1e and different SNR values should be added.

2. It is not very clear how the number of iterations is fixed in the eGRL algorithm, even though the authors mention 10-20 from empirical observations (line 595) and Suppl. fig. 4 shows some metric evolutions with quite different results. In the simulations 20 iterations are used (Fig. 1) while the speed comparisons (with SVD for instance) uses 10 iterations.

3. In the retrieval algorithm, the authors mention an initial estimation using a uniform angular distribution, while it could probably be interesting to start from the distribution obtained form the SVD which is a fast process. Can the authors comment on the choice of this initial distribution and how it affects the reconstruction ?

4. It is not clear which one of the quantities ‘generalized fractional anisotropy’ (GFA) (line 757) and ‘order parameter’ (OP) (line 760) used in the work are measures of the ‘anisotropy’ of the distribution, which seems to be similar to the usually used ‘molecular order’ parameter to give a quantitative measure of the strength in anisotropy of the distribution. Order parameters or equivalent disorder angular metrics have been used in other works (refs. 49 to 51). Is a higher GFA/OP a signature for a higher order and what makes OP values become negative (as seen in a few graphs) since all angles are measured with a modulo 2pi ?

5. In line 241, the authors explain the inferior performance of the SVD algorithm by its use of Gaussian noise and some regularization. It is not clear however in which noise regime the current experiments are done, considering the possibily high number of photons. The authors also chose to show systematically very strong differences obtained between the eGRL and SVD approaches, however their previous work seemed to be entirely based on this approach. It would be interesting to give an idea of the context in which SVD can still be used, considering its advantage in speed.

6. In Fig 3c, it is not clear why the cell nucleus appears entirely in blue color. The authors
do not seem to give a possible explanation (line 275), however it would be good to have one since this is quite puzzling.

7. In the results shown in Fig 3, it would be good to see, in addition with the orientation information, a clearer quantification of the order parameter (OP) obtained, showing images of OP. Similarly in Fig. 4 i,j: some profiles of OPs are given but it would be clearer to see an image of it (similarly as in Fig. 4e).

8. In Line 344, Ref 47 is cited to describe effect of actin alignment in cells, however this does not seem to be the most appropriate illsutration as this paper is rather focussed on the extra cellular matrix alignment. The observed loss of OP could be rather due to the occurrence of a mixture of actin filaments populations, both from the stress fiber bundles and a more isotropic surrounding. Can the authors comment on this possible mixture of populations ?

9. In Lines 436 to 459, the section addressing cell membranes labelled with FM1-43 shows GFA maps and not anymore OP maps, is there a reason for this and why not use the same quantity to quantify such order parameter ? Also there is no clear explanation given for the effect of GFA changes close to the coverslip.

10. In Fig. 5, it would be nice to also display GFA maps, as the degree of order is as important as orientation.

11. Fig. 5iii shows an interesting twisting behavior : how is this observation reproducible ? it would be good to know if this has been observed on several instances or just this specific cell. Can this observation be related to the dynamic behavior of actin at the edge of the cell, involving nucleation/capping and polymerization mechanisms ?

12. In Line 703, the calibration procedure seems to be only a recentering of phases of known polarization curves, however distorsions such as ellipticity frequently occur in polarized microscopy. How is this measured and compensated for ?

13. Despite the high quality of the results obtained, the examples shown are very close to the previously published work by the authors on the same technique and they do not entirely tackle the practical context of light sheet microscopy. Could the authors show the added value of the method by addressing dynamic tissue samples generally measured with this method (ex. drosophila or c.elegans) ?

Other more minor comments :
14. A lot of images in the supplementary figures (ex. Suppl Fig S15,16) do not have axes which permit to known which direction is used to show the sample. It makes it confusing to known which view angle they represent.

15. The legend of Fig. 1e should explain the representation which uses lines and colors, to make it understandable to the reader in all remaining figures.

16. Some quantities are not defined, which makes it confusing to understand the equations which are manipulated in this work (e.g. in Eq. (37) does not define what is mu, V, U.. we can guess their meaning but it would be nice to state this in a rigorous way).

(Remarks on code availability)
I have not runned the code but I have gone through the information and parts of the code lines: it is very well documented.

Version 1:

Reviewer comments:

Reviewer #1

(Remarks to the Author)
The authors have satisfactorily addressed my relatively minor comments. I recommend it for publication.

(Remarks on code availability)

Reviewer #2

(Remarks to the Author)
The authors have adequately addressed the points brought in my initial review, the answers are very thorough and all additional graphs are very useful.

(Remarks on code availability)

Open Access This Peer Review File is licensed under a Creative Commons Attribution 4.0 International License, which permits use, sharing, adaptation, distribution and reproduction in any medium or format, as long as you give appropriate credit to the original author(s) and the source, provide a link to the Creative Commons license, and indicate if changes were made.
In cases where reviewers are anonymous, credit should be given to 'Anonymous Referee' and the source.
The images or other third party material in this Peer Review File are included in the article's Creative Commons license, unless indicated otherwise in a credit line to the material. If material is not included in the article's Creative Commons license and your intended use is not permitted by statutory regulation or exceeds the permitted use, you will need to obtain permission directly from the copyright holder.
To view a copy of this license, visit https://creativecommons.org/licenses/by/4.0/

Response to Referees

Manuscript #:
NCOMMS-26-002418-T

Title:
Efficient spatio-angular reconstruction enables high-fidelity mapping of six-dimensional structures and dynamics with polarized fluorescence microscopy

We thank the Editor and Reviewers for their careful evaluation of our manuscript and for their constructive, insightful feedback. We have thoroughly considered each comment and revised the manuscript accordingly, and we believe the concerns raised have been fully addressed. Our detailed, point-by-point responses (in blue text) are provided as below. All modifications and newly added text are highlighted (in red text) in the revised manuscript and supplementary materials.

Content:

Reviewer 1 2
Reviewer 2 10

Reviewer 1

The authors extend their method for simultaneous 3D spatial and orientational fluorescence microscopy (reported previously in Chandler et al., PNAS 2025) by introducing an efficient generalized Richardson-Lucy (eGRL) deconvolution method to greatly improve accuracy in object estimation over the previous SVD method without much penalty in computing time and memory. The significance of this result is made apparent by the plethora of simulated and experimental demonstrations provided. It will pave the way for wider adoption of their technique. Moreover, the generalization of the RL algorithm to dimensions >3 seems significant to me from an image processing perspective.

In short, the manuscript is a tour de force and it deserve to be accepted with minimal revisions. I have only very minor questions and comments:

RE 1: We thank the reviewer for recognizing the significant technical advantages of eGRL in simultaneous 3D spatial and orientational estimation, and we are pleased that the reviewer appreciates its potential for improved image processing.

• Can the authors clarify their enumeration of "six dimensions" referred to in the title and abstract? 3 spatial + 2 orientation = 5 dimensions. In other works on orientational imaging they also quantify the "wobble angle", which I understand to be the 6th dimension in those papers. Of course, in the current work they go far beyond wobble angle to characterize the ODF in terms of (I think) 1+5+9 = 15 spherical harmonic coefficients.

RE 2: We thank the reviewer for pointing out the ambiguity in our original description.

In conventional polarization fluorescence microscopy, the target is typically parameterized by three spatial coordinates (x, y, z) and a single orientation described by two angular variables (θ, φ), resulting in a 5D representation (i.e. with 3 coordinate variables and 2 orientation scalar angles), or sometimes an additional metric, order of molecule alignment [1].

[1] Brasselet, S. and Alonso, M. A. Polarization microscopy: from ensemble structural imaging to single-molecule 3D orientation and localization microscopy. Optica 10, 1486-1510 (2023)

In the contrast, our method does not estimate only a single average orientation vector at each voxel. Instead, it recovers a full orientation distribution function (ODF): a spherical function

represented as a surface, where the radius along each direction is proportional to the number of dipoles in the measurement volume oriented in that direction.

This distinction is important. An average-orientation representation assigns only one vector to each spatial voxel, i.e., a ***single*** orientation described by (θ, φ); whereas in our manuscript, an ODF assigns a distribution over all possible orientations, i.e., ***a series of*** (θ, φ) orientations with a radius r (or weight) along each orientation. So, there is an additional r variable in addition to coordinate variables (θ, φ), and this orientation observation is fully angular 3D.

That said, our method produces a spatio-angular representation: a 3D spatial volume combined with an orientation distribution represented by a 3D sphere. And as the reviewer says, for compactness, the ODF can also be characterized using spherical harmonic coefficients; in our implementation, 15 coefficients are used to describe the local ODF at each voxel. To make it comply with others in this field, we think it reasonable to leave our method as 6D imaging, in terms of 3D space and 3D angular space.

Also to avoid confusion, we have clarified this point in the revised manuscript, Lines 80-84 in Introduction:
"This framework can obtain both the 3D density and 3D orientation distributions of fluorophores by capturing the fluorescence emissions under polarization modulation. Specifically, the spatio-angular distribution is parameterized by three spatial coordinates (x, y, z), as well as two angular variables (θ, φ) and a radius (r) defining the proportion of molecules along each particular 3D orientation."

We realized that in this fast-developing field of polarization imaging and orientation detection, the definition of imaging dimensions may vary depending on coordinate variables or the number of image scalar values, we has now added an additional **Supplementary Note 3 "Clarification on the dimensions of spatio-angular imaging"** to clarify our claim of "6D" imaging. While we think this note is helpful to the readers, we also defer to the reviewer and editor if they feel that this note could potentially cause misunderstandings and we are open to their further feedback.

• Line 151: "we noticed" should probably be "we note".

RE 3: We thank the reviewer for pointing this out which now has been corrected in Line 154 on Page 4.

• Line 153: "The microscopy" should be "The microscope".

RE 4: We thank the reviewer for this correction which is now revised in Line 156 on Page 4.

• Line 445: "orientational selectivity consistent" should probably be "consistent orientational selectivity".

RE 5: We thank the reviewer for this correction that we have corrected in Line 463 on Page 11.

• Can the authors clarify whether they are coining the term "fluorescent lake"? I haven't heard that before. I think they just mean it to refer to a featureless sample.

RE 6: We agree that this term should be clarified by using more standard terminology. As noted by the reviewer, "fluorescent lake" refers to a spatially and angularly uniform sample, specifically here, "fluorescent lake" refers to a homogeneous fluorescent slide.

We now revised the subscripts and descriptions in Lines 734-738 on Page 19 and Lines 842-848 on Pages 22-23:

"After obtaining the actual polarization angle, we generated the system dipole PSF and simulated the ideal average data $g_{p,v}^{(\mathrm{isoSample})}$ corresponding to a featureless and uniform fluorescent slide under the same instrument configuration. The raw data $g_{p,v}^{(\mathrm{raw})}(\mathbf{r})$ could be corrected by the following form:

$$g_{p,v}(\mathbf{r}) = g_{p,v}^{(\mathrm{raw})}(\mathbf{r})\frac{g_{p_0,v}^{(\mathrm{cal})}}{g_{p,v}^{(\mathrm{cal})}}\frac{g_{p,v}^{(\mathrm{isoSample})}}{g_{p_0,v}^{(\mathrm{isoSample})}}$$

"

"In calibration, the average response corresponding to imaging a spatially and angularly uniform sample with polarization state p *and view* v is calculated to be $g_{p,v}^{(\mathrm{isoSample})}$. We assume that fluorophores in the beads are randomly oriented, so there's merely a global intensity change between the images under polarized and non-polarized illumination. Hence, we scaled the overall intensity of non-polarized beads images by the factor calculated from calibration, to obtain synthetic beads images that would mimic the measurement from polarization p and view v:

$$\mathrm{bead}_{p,v}(\mathbf{r}) = \mathrm{bead}_v(\mathbf{r}) \times \frac{g_{p,v}^{(\mathrm{isoSample})}}{g_{p_0,v_0}^{(\mathrm{isoSample})}}$$

"

• Line 755: Generalized fractional anisotropy is a biological metric"—what is meant by "biological metric"? I think GFA comes from diffusion MRI so maybe it would be more precise to say this along with a citation.

RE 7: We appreciate the reviewer's suggestion and input. We now have revised the text and added citations to clarify its origin in Lines 786–787 on Page 21:
"Generalized fractional anisotropy is a scalar metric[66,67] that quantifies the anisotropy of one single ODF, indicating how oriented the dipoles are in one voxel."

[66] Tuch, D. S. Q-ball imaging. Magnetic Resonance in Medicine 52, 1358–1372 (2004).
[67] Cheng, J. & Basser, P. J. Director Field Analysis (DFA): Exploring Local White Matter Geometric Structure in Diffusion MRI. Medical Image Analysis 43, 112–128 (2018).

• Line 785: "well-designed" should probably be "well-defined".

RE 8: We thank the reviewer's correction which we have incorporated in Line 813 on Page 22.

• In Supplementary Fig. 2 caption they should add a statement on the SSIM that appears in the figure.

RE 9: We thank the reviewer for pointing out this omission. We now have added the statement in caption of **Supplementary Fig. 2**.

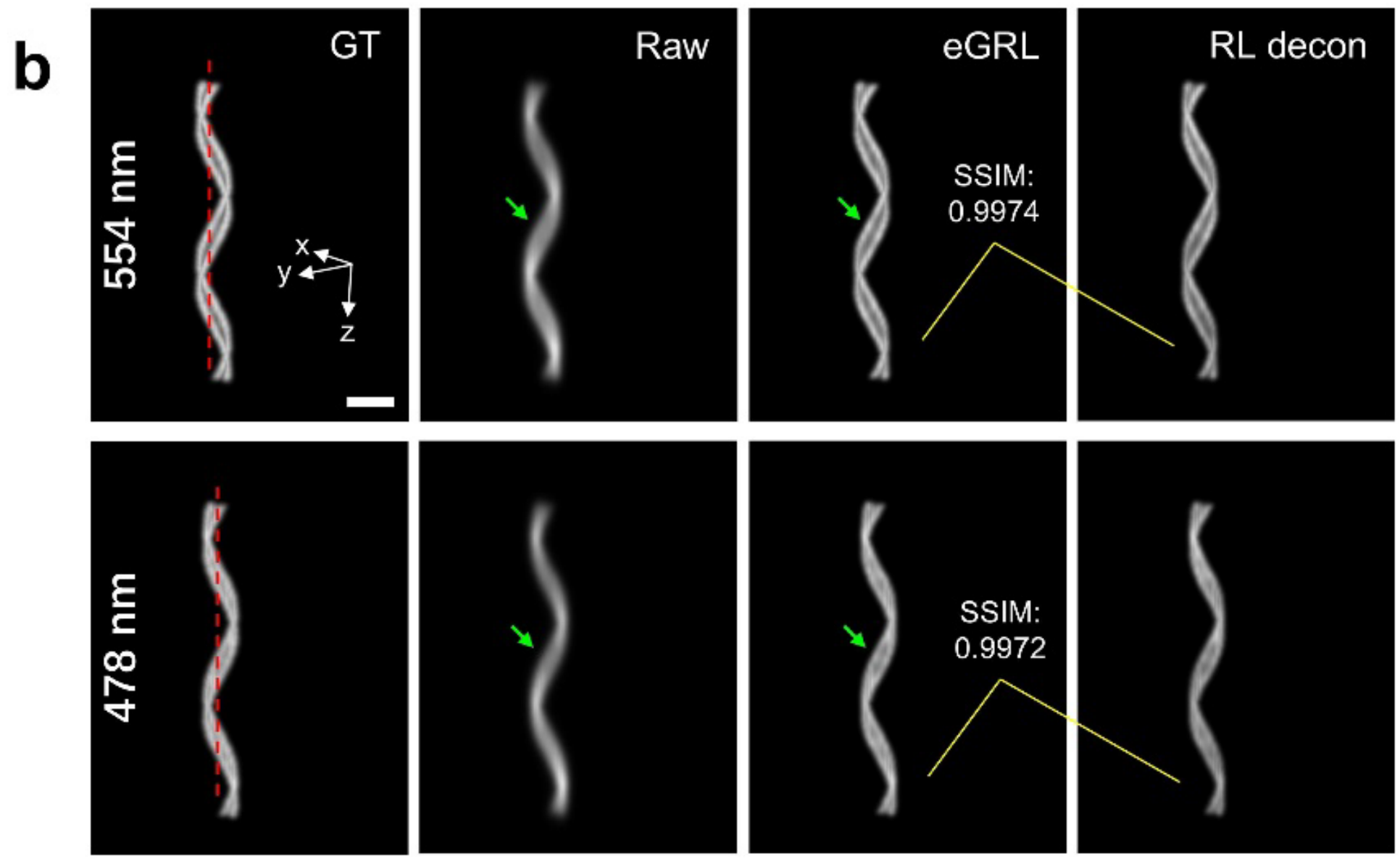

Revised **Supplementary Fig. 2b, eGRL produces high fidelity estimates of oriented phantom structures. b)** Density maps of the double-helix phantoms (from left to right): ground truth (GT),

raw data (average of all polarization measurements), the eGRL reconstruction (20 iterations), and the deconvolved image (RL deconvolution from the averaged raw data, 20 iterations). The SSIM values between the intensity maps obtained from eGRL reconstruction and RL deconvolution are annotated above the figures, demonstrating their high similarity in structural restoration.

• In Supplementary Fig. 4d the OSIM seems to asymptote to something below 1 (around 0.8). Why?

RE 10: We appreciate the reviewer's careful reading. While the overall trend of the curves reflects the similarity between GRL and eGRL, we agree that the reported values may be a bit confusing and could potentially lead to misunderstanding. We now have identified that this issue originates from the underlying definition of the metric OSIM, which lacked proper normalization. We have therefore revised the OSIM formulation and now define it as one minus the mean squared error (MSE) between two ***normalized*** ODFs.

In addition, we realized that we had another metric ONCC in the manuscript to quantify the orientation accuracy, which is redundant. To ensure consistent and reliable quantification, we now use the revised OSIM as the sole metric for evaluating ODF differences throughout the manuscript and have removed or replaced ONCC by OSIM as appropriate.

We revised and improved the definition of OSIM in **Methods**, Lines 801-804 on Page 21:
"The orientational similarity index measure (OSIM) is a newly defined metric, calculated as one minus the mean squared error of each angular component in two normalized ODFs, to quantify the average difference in ODF shape across the entire volume:

$$\mathrm{OSIM} = 1 - \frac{1}{\mathrm{card}(\mathbf{r})}\sum\nolimits_{\mathbf{r}} \frac{1}{\mathrm{card}(\hat{\mathbf{s}})}\sum\nolimits_{\hat{\mathbf{s}}} \left[\frac{f_{\mathrm{GT}}(\mathbf{r},\hat{\mathbf{s}})}{\max\limits_{\hat{\mathbf{s}}}[f_{\mathrm{GT}}(\mathbf{r},\hat{\mathbf{s}})]} - \frac{f_{\mathrm{O}}(\mathbf{r},\hat{\mathbf{s}})}{\max\limits_{\hat{\mathbf{s}}}[f_{\mathrm{O}}(\mathbf{r},\hat{\mathbf{s}})]}\right]^2$$

where f_{GT} and f_{O} are the spatio-angular distribution of ground truth and eGRL output; $\mathrm{card}(\mathbf{r})$ and $\mathrm{card}(\hat{\mathbf{s}})$ are the number of elements in $\mathbf{r}$ and $\hat{\mathbf{s}}$. "

With the new OSIM, the corresponding quantification results are updated as follows – and ***we'd like to clarify that the updated OSIM values align with the statements/conclusions in the manuscript and do not change any interpretations of eGRL performance***:

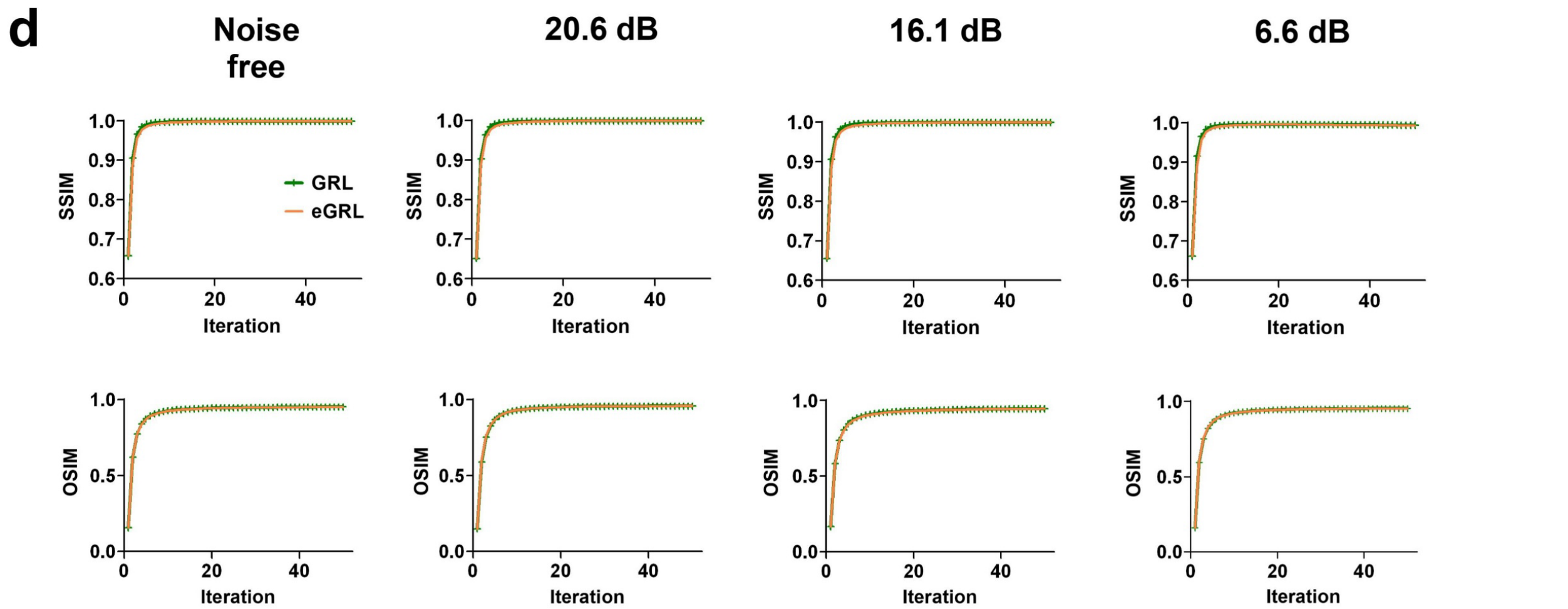

Revised **Supplementary Fig. 5d**, **eGRL and GRL provide similar image quality as assayed on synthetic data. d)** SSIM and OSIM (ODF similarity index measure. See **Methods**) analysis of the ODF maps between the reconstructions and ground truth. Note extreme similarity between eGRL and GRL.

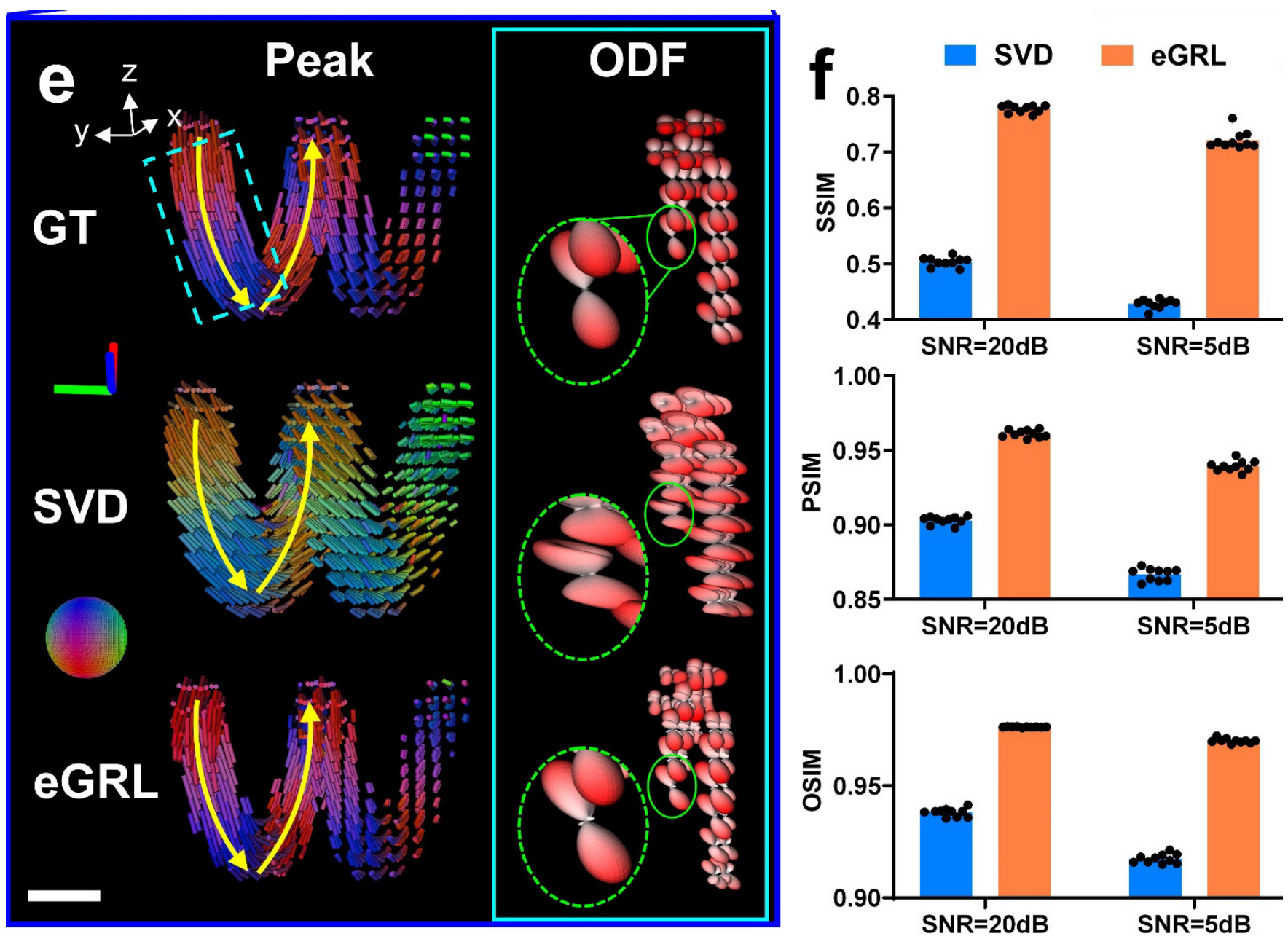

Revised **Fig. 2e, f**, **Spatio-angular image reconstruction from simulated data of different polarized fluorescence microscopes. f)** Quantitative analysis of the reconstruction performance on dual-view light sheet data (diSPIM) with two different noise levels (SNR=20 dB and SNR=5 dB), with SSIM, PSIM and OSIM quantifying the reconstruction accuracy of density, peak orientation, and ODF, respectively. The black dots represent 10 individual repeated measurements.

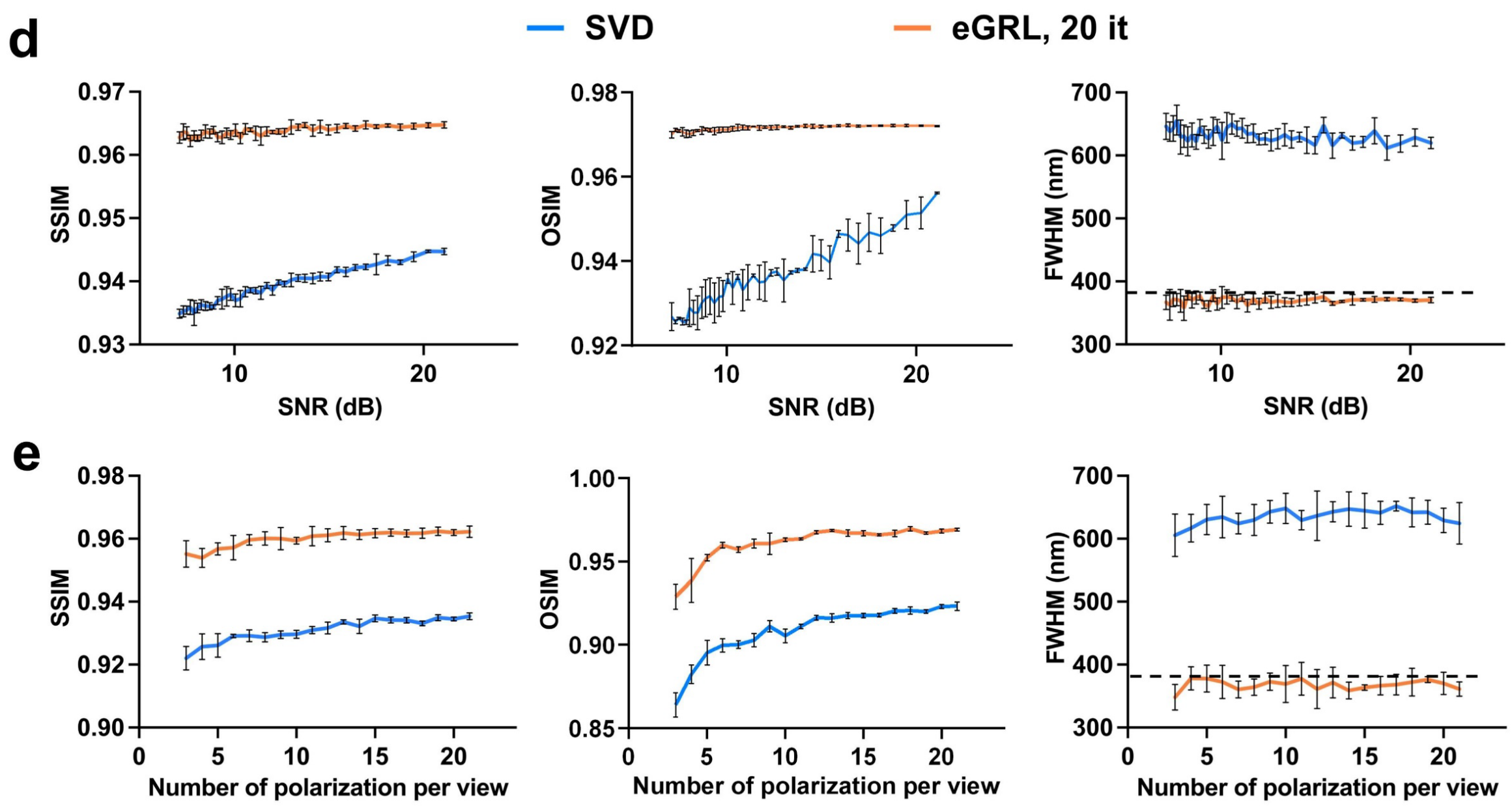

Revised **Supplementary Fig. 9d, e, Performance of eGRL and SVD on synthetic data with different noise levels and different number of polarization modulations. d-e)** Evaluation metrics (SSIM, OSIM, and FWHM) vs. input data SNR and number of polarization modulations curves are plotted for the comparison of eGRL and SVD.

• In Supplementary Fig. 12a are the labels for “c” and “d” swapped?

RE 11: We thank the reviewer for uncovering this error. We now have corrected this.

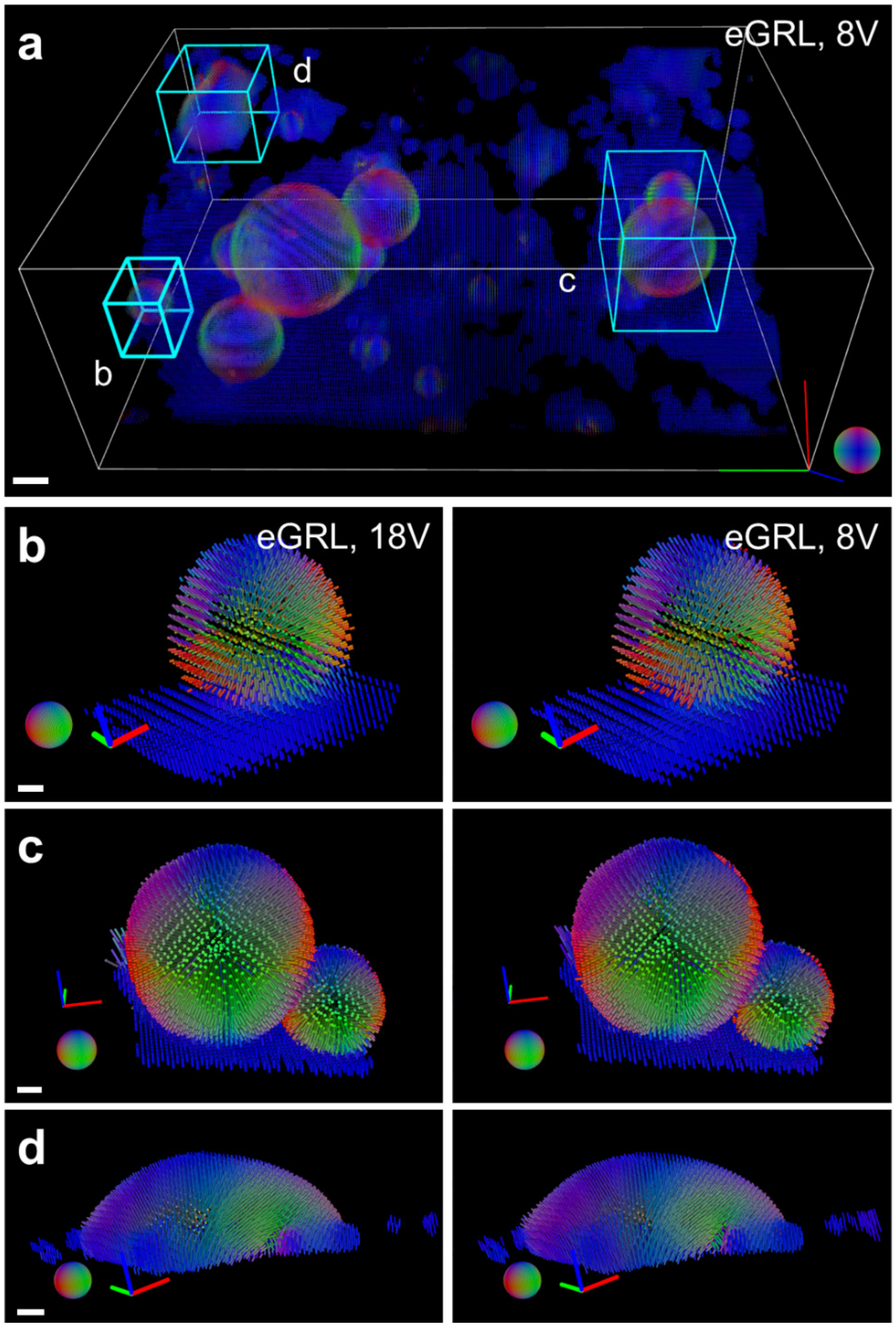

Revised **Supplementary Fig. 13**, **Performance of eGRL with a reduced number of polarization modulations on GUV sample. a)** eGRL reconstruction of FM1-43 labelled GUVs used only 8 polarization modulations (Scheme 2, 8V. See **Supplementary Table 2**), by contrast to the results obtained using a full complement of 18 modulations (Scheme 1, 18V. **See Supplementary Table 2**) as shown in **Fig. 2a**. **b-d)** Comparisons of eGRL reconstructions using both 18 and 8 polarization modulations across the 3 cyan-labelled cubic regions specified in **a**. **Scale bars**: **a** 5 µm, **b** 1 µm, **c**, **d** 2 µm.

Reviewer 2

In this work, the authors develop a method to image three-dimensional orientation properties of fluorophores in lipid membranes and cell samples, using volumetric imaging microscopy. Polarization resolved light sheet imaging, in particular using a polarized dual-view light-sheet system, leads to a full 6D spatio-angular information with reliable reconstruction. The work is mostly concentrated on the improvement of the data processing method as compared to a previously published work by the authors (Proceedings of the National Academy of Sciences, 2025). This method, based on an efficient generalized Richardson-Lucy (eGRL) algorithm, makes a convincing use of spherical harmonics decompositions, exhibits high quality results and a clear advantage over previous approaches in terms of processing speed and efficiency. The theoretical analysis is based on well-chosen mimicking phantom objects, whose shape and orientational distributions can be challenging to measure in 3D. The work is sound and well presented despite a few unclear aspects as detailed below. The robustness of the method and its increased speed opens the way to measurements of structures in 3D in live samples, which is a novelty.

RE 12: We thank the reviewer for summarizing our work and recognizing the significance of eGRL in measuring 3D spatial structures and molecular orientations in live samples.

1. The theoretical study of Fig. 1e displays a comparison between the ground truth on helical patterns and their reconstruction. The message given from it and other theoretical figures throughout the work is rather qualitative, it is difficult to see how much the reconstruction deviates from the ground truth. For instance, while the color evolution along the helices seem to be nicely reproduced, some lines are clearly oriented differently and of different lengths than the ground truth: it would be good to have a more quantitative idea of what these differences represent. Monte Carlo simulations could be used to quantify both accuracy and precision on all retrieved parameters, their inter-dependence and their sensitivity to signal to noise levels. Similarly, order parameters values can be very low but their precision is unknown (for ex. in Suppl Fig S15-iii). Graphs showing accuracy and precision values for both orientation and anisotropy (GFA or OP) for different kinds of phantoms displayed in Fig. 1e and different SNR values should be added.

RE 13: These comments and suggestions on quantifying accuracy and precision are very insightful and helpful, please see our responses:

1) Previous quantifications of the accuracy

We would first like to clarify the original purpose of the result shown in **Fig. 1e**. It was intended to provide readers with an initial impression of the simultaneous estimation of structure and orientation; therefore, we did not include detailed quantitative analysis in this figure. More comprehensive quantification was mainly in **Fig. 2** and the corresponding supplementary figures with richer structures and orientations. We evaluated performance under varying SNR levels and different numbers of polarization modulations (**Fig. 2f**, **g** and **previous Supplementary Fig. 4**, **8**, **now Supplementary Fig. 5**, **9**) using orientation histograms, SSIM, PSIM, OSIM, and FWHM, all benchmarked against the ground truth.

That said, we agree that, for **Fig. 1e**, we did not comprehensively analyse the accuracy and precision of all retrieved parameters, their interdependence, and their sensitivity to SNR, but we have now included them in the revised manuscript, as suggested by the reviewer.

2) Accuracy and precision via Monte Carlo simulation

Also following the reviewer's suggestion, we have evaluated the bias and variability of the estimated parameters, including orientation (θ, φ) and anisotropy (GFA). We swept through all values of the parameters $\theta \in [0, 90°]$ and $\mathrm{GFA} \in [0.05, 0.9]$ with a step of 2° and 0.02, respectively under $\varphi = 10°$ and SNR=20 dB or 2dB. We repeated each parameter combination 350 times to evaluate the accuracy (mean value, i.e. Δ_φ, Δ_θ, Δ_{GFA}) and precision (standard deviation value, i.e. σ_φ , σ_θ , σ_{GFA}) of eGRL estimation. We also summarize all the 1620 combinations × 350 repeats via histograms, to show how the parameter retrieval errors are distributed in general.

We now have included the analysis in the new **Supplementary Fig. 4** and added texts in Lines 202-210 on Pages 5-6:

"In addition, Monte Carlo simulations[2,29] were performed to evaluate the accuracy and precision of orientational parameter retrieval under different noise conditions (**Supplementary Fig. 4**). At high SNR (20 dB), the estimation errors of azimuthal angle φ, polar angle θ and generalized fractional anisotropy (GFA) were tightly centered around zero, with low standard deviations of 1.07°, 1.19°, and 0.009, respectively. In contrast, under low SNR (2 dB), the error distributions became broader, with the standard deviations increasing to 3.67°, 4.21°, and 0.02. By systematically varying orientation and anisotropy, we found that orientational reconstruction is robust over a wide range of parameters although some larger estimation uncertainty occurred in low-GFA regions and under extreme angular conditions."

[2] Zhang, O. et al. Six-dimensional single-molecule imaging with isotropic resolution using a multi-view reflector microscope. Nat. Photon. 17, 179–186 (2023).

[29] Senthil Kumar, C. S. et al. 4polar3D single molecule imaging of 3D orientation in dense actin networks using ratiometric polarization splitting. Nat Commun 17, 4246 (2026).

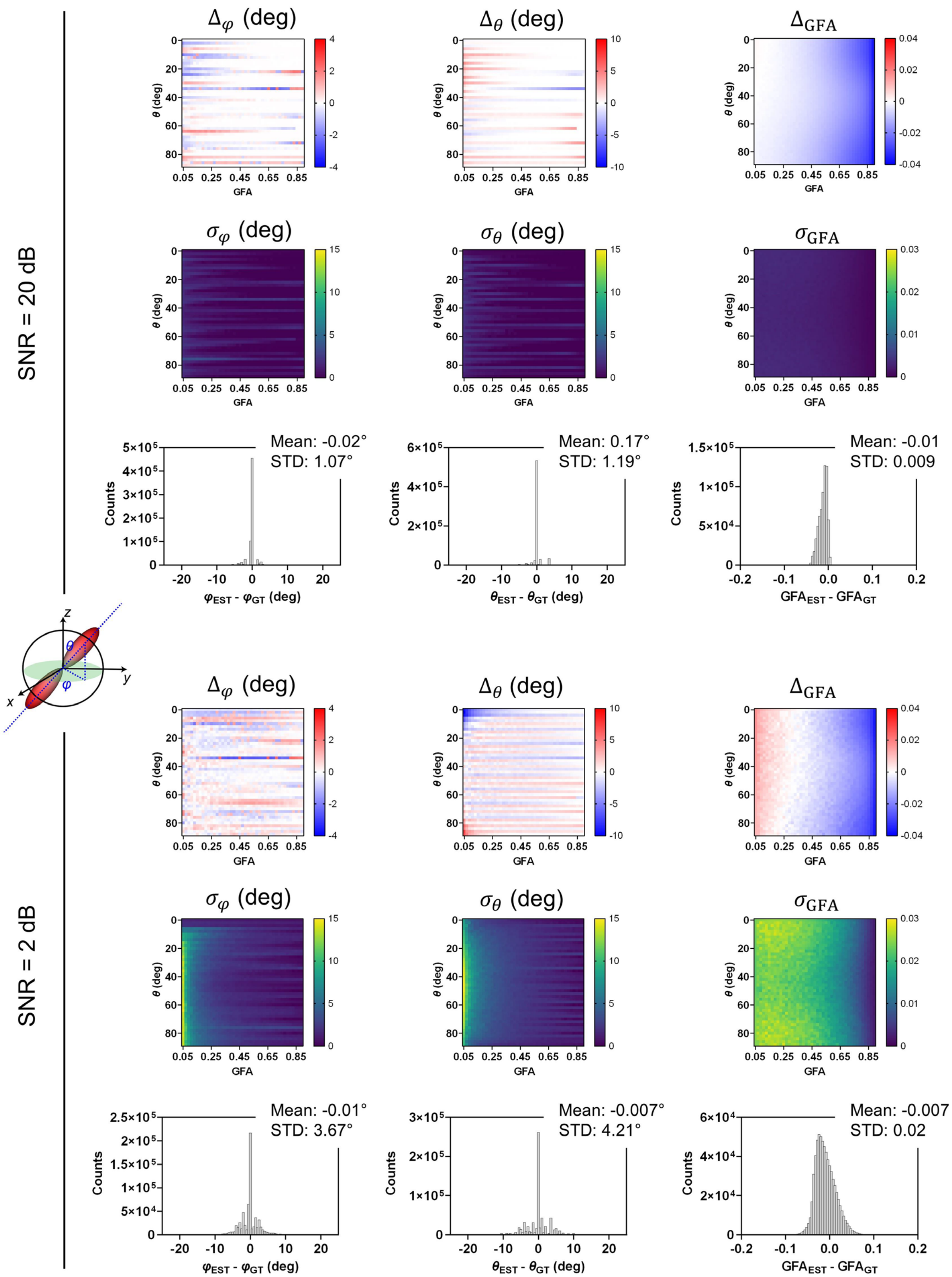

New **Supplementary Fig. 4, Accuracy and precision analysis of orientational parameter estimation via eGRL under different SNR conditions.** Monte Carlo simulations conducted with: 350 ODFs per image, 1620 images, 20 dB or 2 dB Poisson noise. Schematic illustration of the ODF described by the azimuthal angle φ, polar angle θ and GFA. Simulations were performed by varying θ (2° step from 0° to 90°) and GFA (0.02 step from 0 to 0.9) while fixing φ to 10°. 1st, 2nd, 4th and 5th row, heatmaps showing the mean estimation error (Δ_{φ}, Δ_{θ}, and Δ_{GFA}) and the corresponding standard deviations (σ_{φ}, σ_{θ}, and σ_{GFA}) for the recovered azimuthal angle φ, polar angle θ, and GFA. 3rd and 6th row, histograms of the estimation errors for φ, θ, and GFA under two representative Poisson noise conditions (20 dB and 2 dB). The mean and standard deviation (STD) of each distribution are indicated in the upper right corner of each panel.

3) Clarification on the low OP values in previous **Supplementary Fig. 15** (now **Supplementary Fig. 16**)

OP of the ODF describes the average degree of alignment of molecular orientations with respect to a reference direction. We mainly investigated cells grown on two types of nanowire substrates, namely parallel and crossed configurations (**Fig. R1**).

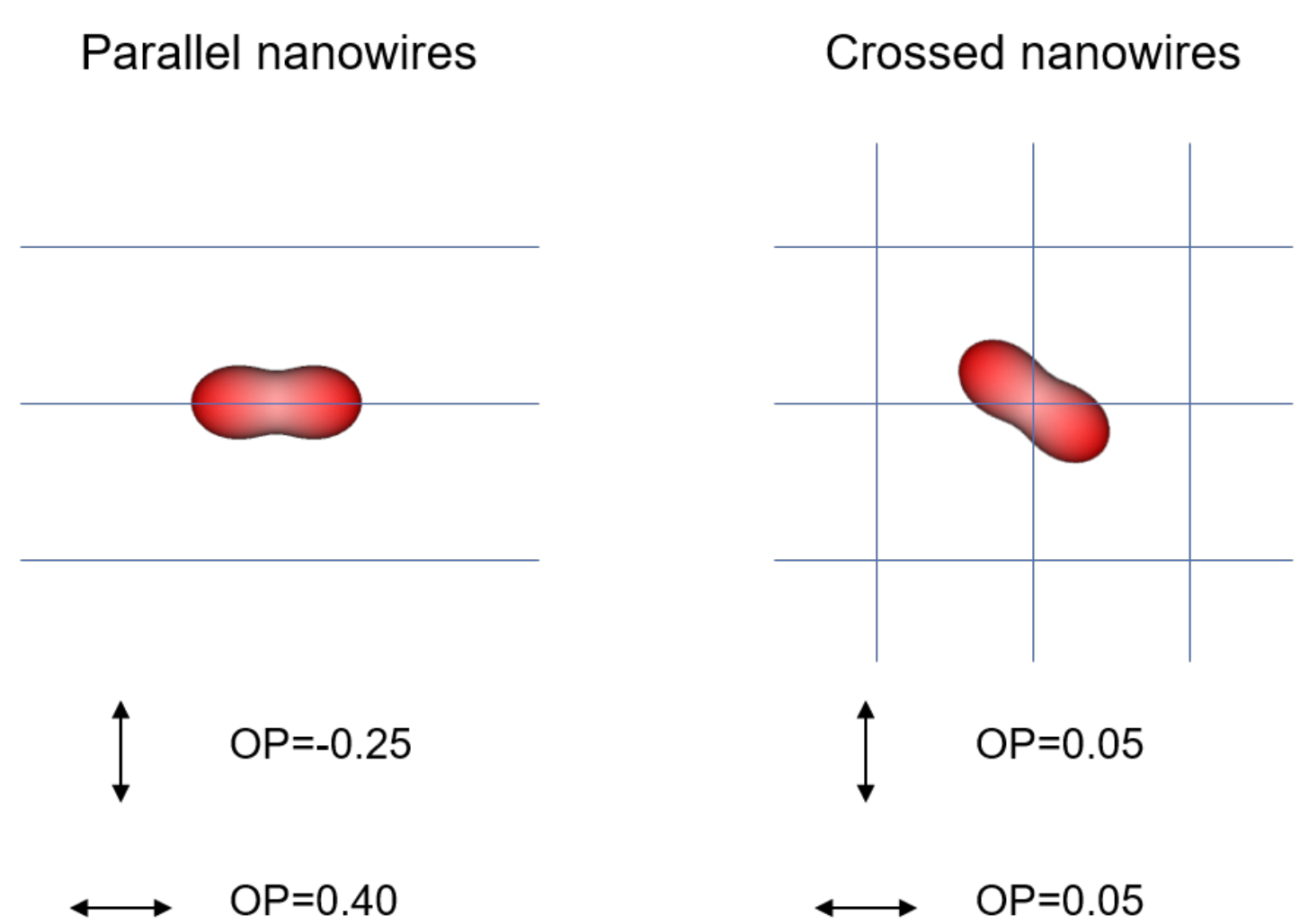

Fig. R1, Schematic illustration of representative ODFs and OPs in parallel and crossed nanowire matrices.

In the case of parallel nanowires (**Supplementary Fig. 16b**), the molecules tend to align along the wire direction, resulting in relatively high OP values (0.2~0.6) along this direction, while the OP along the orthogonal direction remains low. In contrast, for crossed nanowires, particularly in dense wire matrices (**Supplementary Fig. 16c**), actin molecules tend to orient between the

horizontal and vertical directions. As a result, the projected OP values along each of these two directions are very low (-0.01~0.05) in our analysis.

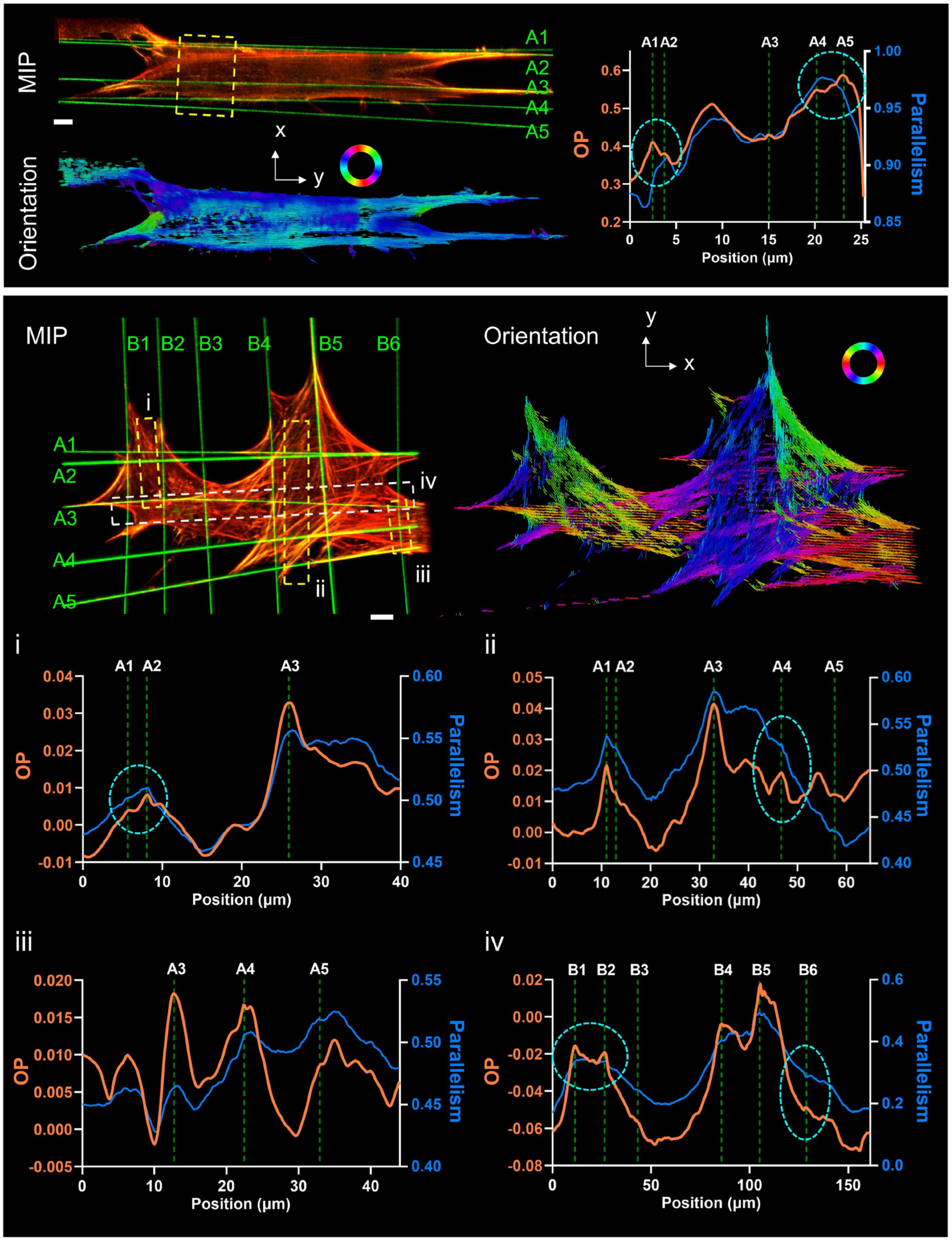

Supplementary Fig. 16b, c, Spatio-angular reconstructions show correlations between the direction of actin molecules and the direction of nanowires upon which the cells are grown. b) Twin cells grown on multiple nanowires in roughly parallel directions. Similar representations and analysis are demonstrated as in **a**. **c)** Extended analysis of the crosshatched actin fibers grown on

crisscrossed nanowires in **Fig. 3g**. Note that our spatio-angular imaging and ODF reconstruction demonstrate explicit patterns of dipole preference, which are not discerned in the intensity images or the average orientation detection by traditional PFMs (see especially those comparisons outlined by cyan dashed circles). Scale bars: 10 µm.

2. It is not very clear how the number of iterations is fixed in the eGRL algorithm, even though the authors mention 10-20 from empirical observations (line 595) and Suppl. fig. 4 shows some metric evolutions with quite different results. In the simulations 20 iterations are used (Fig. 1) while the speed comparisons (with SVD for instance) uses 10 iterations.

RE 14: We thank the reviewer for pointing out the ambiguity of the iteration numbers.

1) We would like to clarify that in the analysis in previous **Supplementary Fig. 4**, we used the unnormalized ODFs to calculate OSIM that led to a large discrepancy between the results, and we now have revised it to the updated OSIM metric in Lines 801-804 on Page 21 (also see details in our response **RE10** to Reviewer #1):
"The orientational similarity index measure (OSIM) is a newly defined metric, calculated as one minus the mean squared error of each angular component in two normalized ODFs, to quantify the average difference in ODF shape across the entire volume:

$$\mathrm{OSIM} = 1 - \frac{1}{\mathrm{card}(\mathbf{r})}\sum_{\mathbf{r}} \frac{1}{\mathrm{card}(\hat{\mathbf{s}})}\sum_{\hat{\mathbf{s}}} \left[\frac{f_{\mathrm{GT}}(\mathbf{r},\hat{\mathbf{s}})}{\max_{\hat{\mathbf{s}}}[f_{\mathrm{GT}}(\mathbf{r},\hat{\mathbf{s}})]} - \frac{f_{\mathrm{O}}(\mathbf{r},\hat{\mathbf{s}})}{\max_{\hat{\mathbf{s}}}[f_{\mathrm{O}}(\mathbf{r},\hat{\mathbf{s}})]}\right]^2$$

where f_{GT} and f_{O} are the spatio-angular distribution of ground truth and eGRL output; $\mathrm{card}(\mathbf{r})$ and $\mathrm{card}(\hat{\mathbf{s}})$ are the number of elements in $\mathbf{r}$ and $\hat{\mathbf{s}}$."

And the corresponding quantifications, demonstrating the convergent tendency:

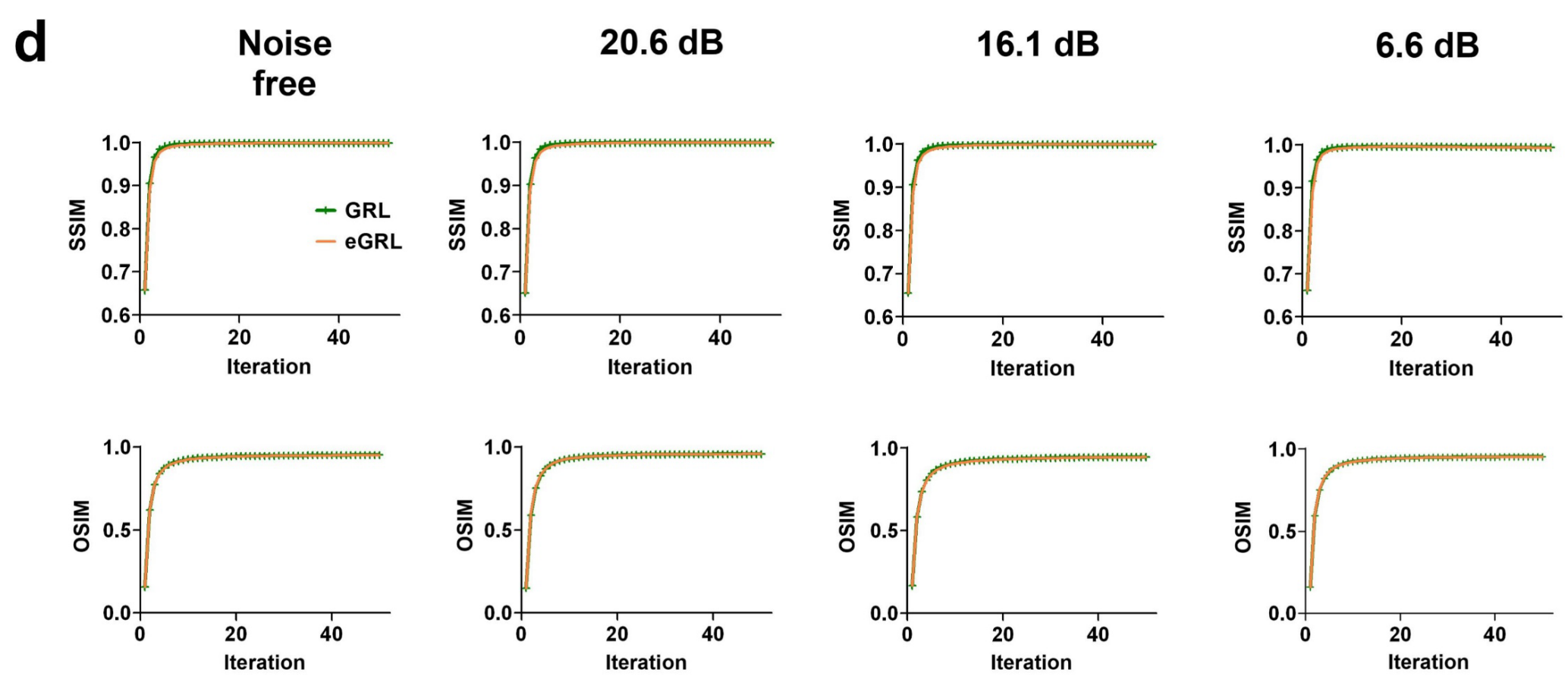

Revised **Supplementary Fig. 5d**, **eGRL and GRL provide similar image quality as assayed on synthetic data. d)** SSIM and OSIM (ODF similarity index measure. See **Methods**) analysis of the ODF maps between the reconstructions and ground truth. Note extreme similarity between eGRL and GRL.

2) We would also like to clarify that the results obtained with 10 and 20 iterations are highly similar. Specifically, the simulations presented in the revised **Supplementary Fig. 5d** above show that the two settings yield highly comparable results in both structural and orientational evaluations. To further support the analysis, we have additionally quantified the convergence of eGRL iteration on experimental GUV datasets and obtained similar results:

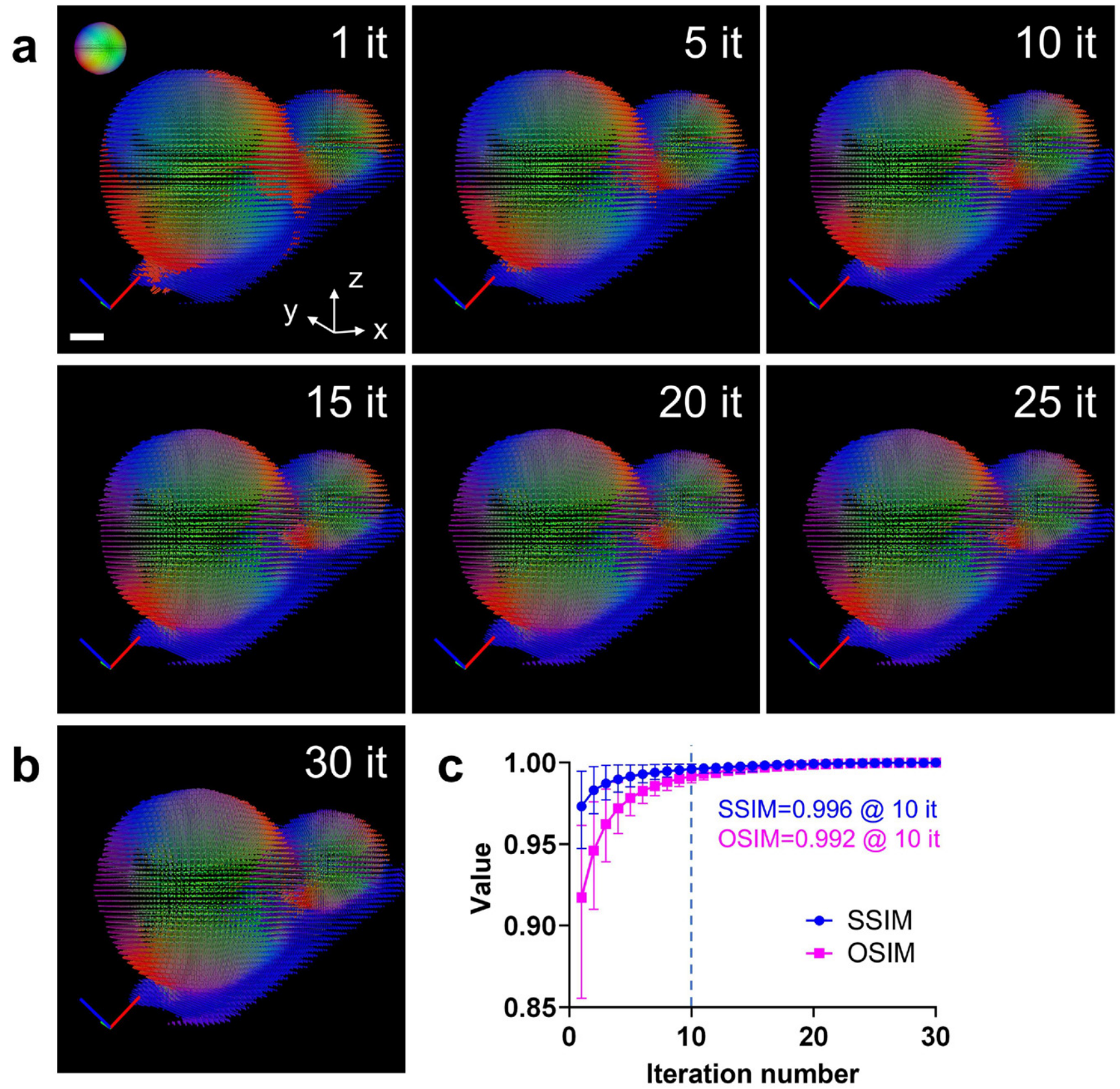

New **Supplementary Fig. 25**, **Iterative convergence analysis of eGRL reconstruction. a)** Representative orientation reconstruction results obtained after 1, 5, 10, 15, 20, and 25 iterations of eGRL. The reconstructed orientation distribution gradually converges with increasing iteration number, while the overall structural features become stable after approximately 10 iterations. **b)** Final reconstruction result after 30 iterations, shown as the reference for convergence evaluation. **c)** Convergence analysis quantified by SSIM and OSIM as functions of iteration number. Error bars indicate the range across the 10 random ROIs. Both SSIM and OSIM approach saturation after approximately 10 iterations, reaching SSIM = 0.996 and OSIM = 0.992 at 10 iterations. Scale bar: 2 μm.

3) Regarding the choice of the stopping criterion for the experimental datasets, all reconstructions were performed using 10 eGRL iterations, as improvement after 10 iterations was marginal but more iterations imply more computational cost. The same setting was also adopted for comparing the computational cost for the SVD-based algorithm.

4) Considering that the stopping criterion is more an experimental recommendation than a hard rule, now we have also softened our language and revised this guideline so that it applies to setups similar to our pol-diSPIM with iteration number range 10-20 based on our simulation and experimental tests, see Lines 633-636 on Page 17:
"We recommend 10-20 iteration alternating dual-view eGRL deconvolution as a good rule of thumb in pol-diSPIM or similar setups to balance processing time and spatio-angular resolution, based on simulations (**Supplementary Fig. 5**) and experience with our experimental datasets (**Supplementary Fig. 25**)."

3. In the retrieval algorithm, the authors mention an initial estimation using a uniform angular distribution, while it could probably be interesting to start from the distribution obtained form the SVD which is a fast process. Can the authors comment on the choice of this initial distribution and how it affects the reconstruction?

RE 15: This is a very interesting point, and we now have tested it on synthetic datasets with randomly distributed GUV phantoms. We compared three initialization strategies, including averaged intensity with uniform angular distribution (noted by Mean), estimated result from SVD (SVD) and, uniform spatial and angular distribution (Uniform). We investigated the convergence over 1~30 iterations.

SVD initialization gives the best starting estimate and therefore achieves higher SSIM and OSIM in the early iterations. But this advantage is limited to the initial stage, as after ~10 iterations, convergence is similar across all initialization strategies.

Inspired by the reviewer's suggestion, we have included the above conclusion in new **Supplementary Fig. 26** and text in Lines 636-640 on Page 17:
"And the initial estimate is given by the average of images in polarization and view channels:

$$E_{0,lm}(\mathbf{r}) = \begin{cases} \sum_{\hat{\mathbf{p}}} i_{\hat{\mathbf{p}}}^{(v1)}(\mathbf{r}) + \sum_{\hat{\mathbf{p}}} i_{\hat{\mathbf{p}}}^{(v2)}(\mathbf{r}), & l = 0, m = 0 \\ 0, & \text{others} \end{cases}$$

We also found that eGRL converges reliably when initialized using the SVD reconstruction or a spatially and angularly uniform structure (**Supplementary Fig. 26**)."

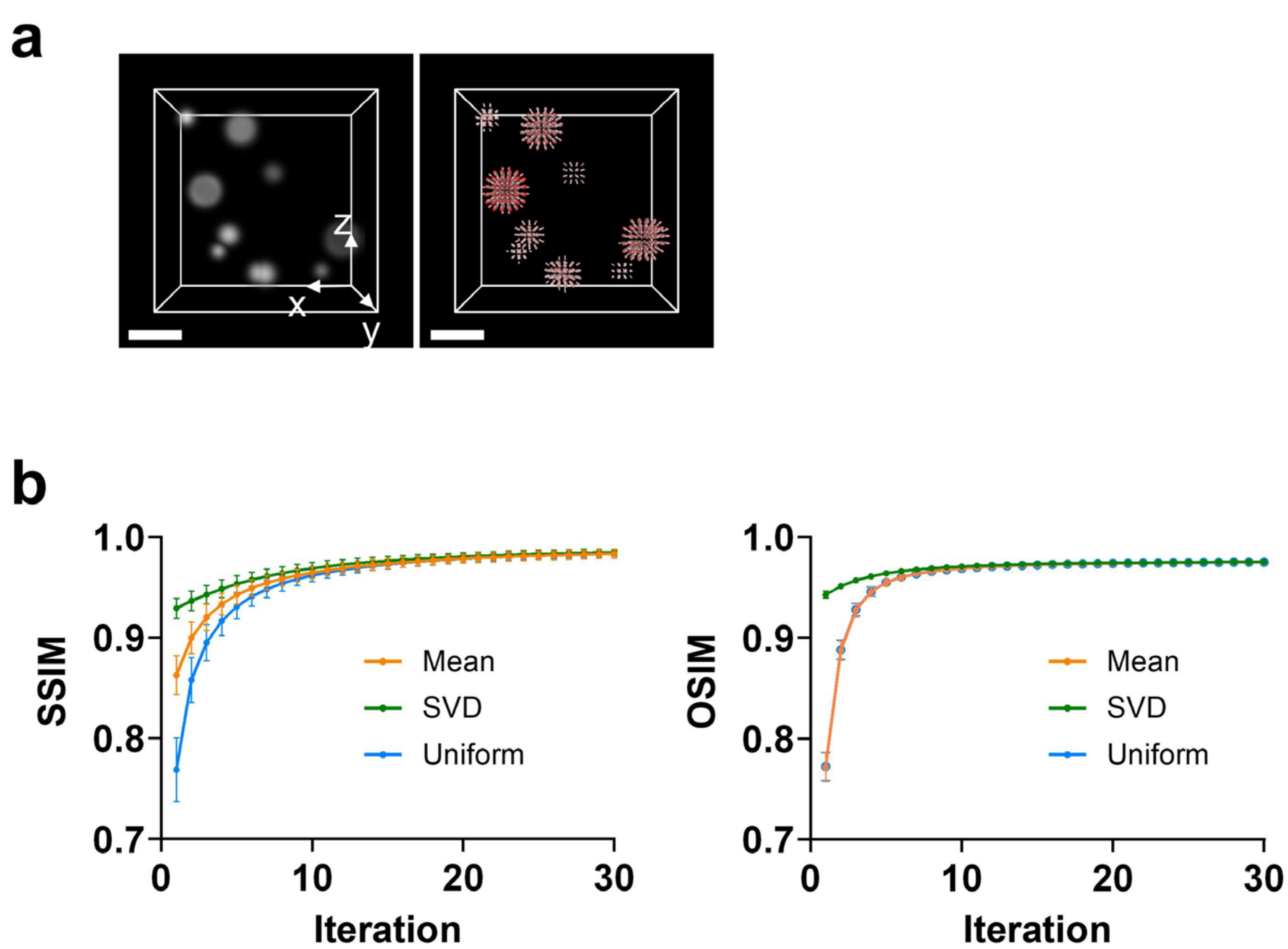

New **Supplementary Fig. 26**, **Comparison of different initialization strategies for eGRL reconstruction. a)** Density and ODF map of the simulated dataset consisting of multiple spherical structures with randomly distributed positions and different sizes. **b)** Comparison of convergence using three different initialization strategies: the mean of the raw intensity data (Mean), the SVD result (SVD), and a spatially and angularly uniform structure (Uniform), quantified by SSIM and OSIM. Error bars indicate the standard deviation across 10 repeated simulations. Note that SVD initialization provides a more accurate starting point during the early iterations while after ~10 iterations, all three initialization strategies show similar convergence behavior and approach nearly identical final performance. Scale bar: 2 µm.

4. It is not clear which one of the quantities 'generalized fractional anisotropy' (GFA) (line 757) and 'order parameter' (OP) (line 760) used in the work are measures of the 'anisotropy' of the distribution, which seems to be similar to the usually used 'molecular order' parameter to give a quantitative measure of the strength in anisotropy of the distribution. Order parameters or equivalent disorder angular metrics have been used in other works (refs. 49 to 51). Is a higher GFA/OP a signature for a higher order and what makes OP values become negative (as seen in a few graphs) since all angles are measured with a modulo 2pi?

RE 16: We thank the reviewer for raising this point that might confuse the readers. Please see our clarifications:

1) The Generalized Fractional Anisotropy (GFA) used in our manuscript is a scalar metric originating from diffusion MRI [1, 2], that quantifies the strength in anisotropy of the distribution. As far as we know, this metric is very similar to 'molecular order' [3].

[1] Tuch, D. S. Q-ball imaging. Magnetic Resonance in Medicine 52, 1358–1372 (2004).
[2] Cheng, J. & Basser, P. J. Director Field Analysis (DFA): Exploring Local White Matter Geometric Structure in Diffusion MRI. Medical Image Analysis 43, 112–128 (2018).
[3] Loison O. et al. Polarization-resolved microscopy reveals a muscle myosin motor-independent mechanism of molecular actin ordering during sarcomere maturation. PLOS Biology 16, e2004718 (2018).

2) Unlike GFA or molecular order, the order parameter (OP) quantifies the extent to which the dipole distribution in the ODF is aligned with ***a specified reference direction***. This direction can be defined arbitrarily and does not necessarily need to correspond to the peak or preferential orientation of the distribution.

For instance, in **Supplementary Fig. 15a**, the ODFs in groups 2 and 4 have the same spindle shape and therefore the same GFA (~0.3), but their peak orientations are orthogonal. Consequently, their alignments with respect to a given reference direction are substantially different, which can be captured by the order parameter but not by GFA alone.

Regarding the sign and interpretation of OP, in group 1, OP=1 indicates perfect alignment along the reference direction; in group 3, OP=0 typically indicates no preferred orientation; and in group 4 and 5, OP<0 indicates that the orientations are preferentially distributed perpendicular to the reference direction.

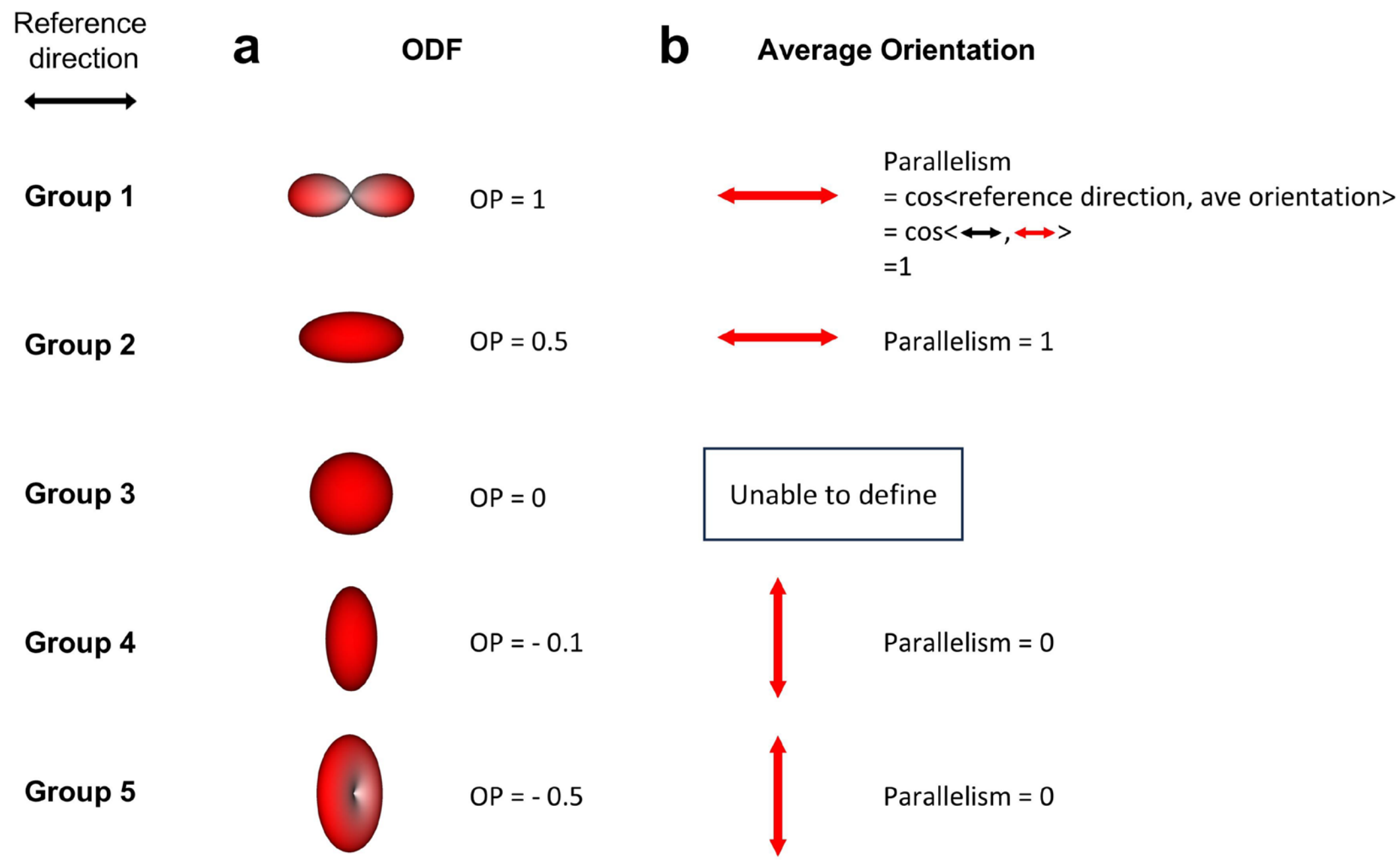

Supplementary Fig. 15a, ODFs and OPs provides a useful assessment of dipoles' preference towards a reference direction. a) The ODF shapes of five different groups of dipoles and the corresponding order parameter (OP) values with respect to the direction shown at upper left (labeled as 'reference direction').

3) We summarize the above clarification in the following table:

	Definition	Interpretation to values
OP	Describes the average degree of alignment of molecular orientations with respect to ***a reference direction***	Larger OP value: molecules better aligned with the reference direction<br><br>Smaller OP value: molecules tend to be less aligned with the reference direction
GFA	Describes the general anisotropy or concentration of the full orientation distribution regardless to any reference	Larger GFA value: A more concentrated and anisotropic distribution<br><br>Smaller GFA value: a more uniform and isotropic distribution
Molecular order	Represented by the angular aperture, describing the spread of molecular orientations	Larger value: broader and less ordered orientations<br><br>Smaller value: more concentrated and ordered orientations

We primarily would like to leave the above table in the response letter (because we don't use Molecular order in the manuscript), but would also defer to the reviewer or editor if we need to add it to the manuscript.

5. In line 241, the authors explain the inferior performance of the SVD algorithm by its use of Gaussian noise and some regularization. It is not clear however in which noise regime the current experiments are done, considering the possibily high number of photons. The authors also chose to show systematically very strong differences obtained between the eGRL and SVD approaches, however their previous work seemed to be entirely based on this approach. It would be interesting to give an idea of the context in which SVD can still be used, considering its advantage in speed.

RE 17: We thank the reviewer for this helpful suggestion to provide a more comprehensive description of the differences between our previous method SVD and the current proposed method eGRL.

1) Although polarization modulation was added to our diSPIM system, we assume that the noise characteristics of the raw measurements are still dominated by Poisson statistics under the current imaging conditions. In our data obtained from ORCA-Flash4.0 cameras, the cellular signal is typically greater than 500 photoelectrons, whereas the background is approximately 50 photoelectrons with an exposure time of 15 ms per plane. This substantial signal-background separation suggests that photon-counting fluctuations remain the primary noise source, while the contribution of other detector-related noise components is comparatively less significant.

2) SVD and eGRL can be regarded as extensions of Wiener filter (was widely used) and RL deconvolution (now more commonly adopted), respectively. RL is more consistent with the Poisson statistics, and generally offers stronger sharpening and detail recovery, although it is computationally more intensive due to its iterative nature. In contrast, the Wiener filter is computationally efficient and numerically stable, but as a fundamentally linear and more conservative restoration method, it is often less effective than RL in recovering high-frequency details and may lead to over-smoothing or information loss if the regularization is not properly chosen.

3) SVD and eGRL inherit the characteristic properties of the two widely used reconstruction frameworks from which they are derived. In the case of SVD, we believe that the bias in orientation estimation is primarily introduced by the regularization, rather than by the assumed noise distribution. For images with a higher input SNR, SVD requires weak regularization, under which it can yield more accurate estimates (**Supplementary Fig. 9d**) that are comparable to those

from ground truth (**Fig. R2**). This is the regime that we believe SVD can achieve its best balance of reconstruction accuracy and computational efficiency.

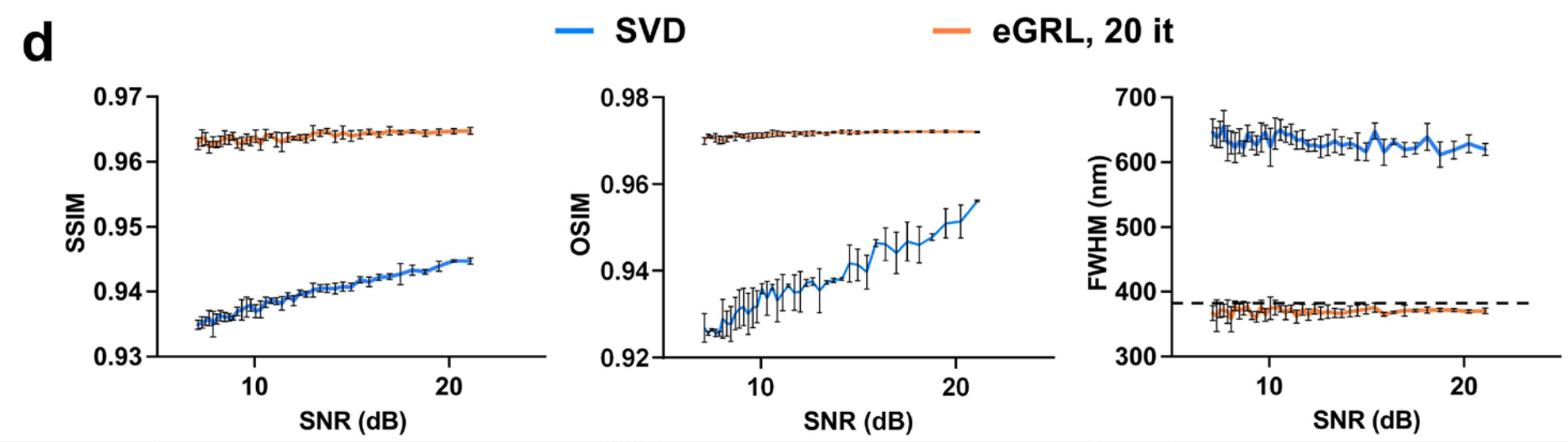

Revised **Supplementary Fig. 9d**, **Performance of eGRL and SVD on synthetic data with different noise levels and different number of polarization modulations. d)** Evaluation metrics (SSIM, OSIM, and FWHM) vs. input data SNR curves are plotted for the comparison of eGRL and SVD.

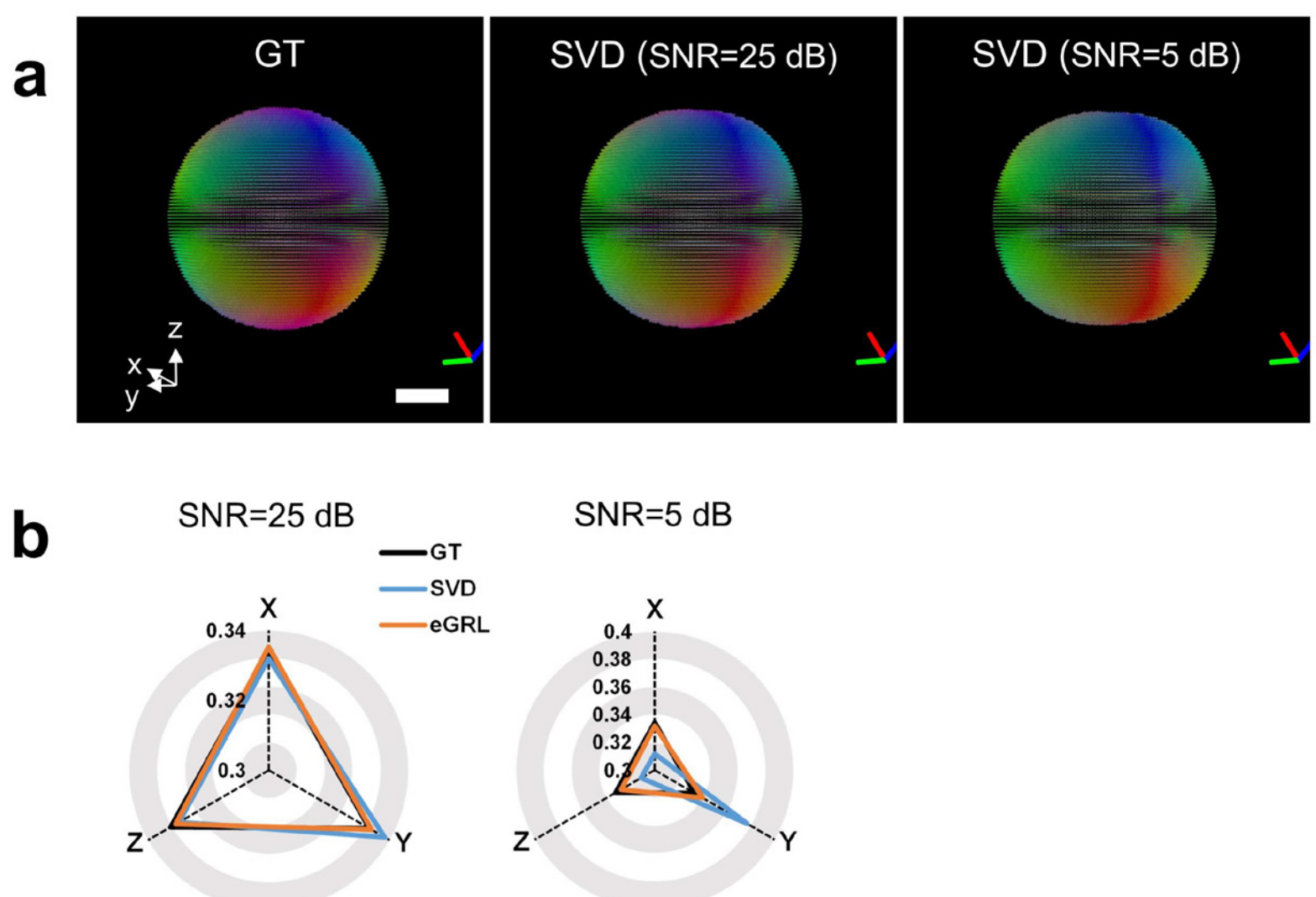

Fig. R2, **SVD performance under different noise levels. a)** Comparison of peak orientations reconstructed from synthetic shell data using SVD under different noise levels, with SNR = 25 dB and 5 dB. **b)** Radar chart showing the proportions of peak orientations assigned to the x, y, and z axes, ranging from 0 to 1. The results highlight the less biased peak-orientation estimation achieved by SVD under high SNR conditions. **Scale bars**: **a** 2 µm.

4) In addition, our simulations indicate that the bias in SVD-based estimation is systematic and associated with the regularization term (**Supplementary Movie 4**, or see montage in **Fig. R3** below), which tends to shift the recovered orientations toward the y axis, rather than being random. Therefore, although SVD reconstruction may not provide fully accurate absolute quantification, we believe that the main conclusions of the previous study remain valid, especially with regard to the observed trends and relative comparisons at high SNR levels.

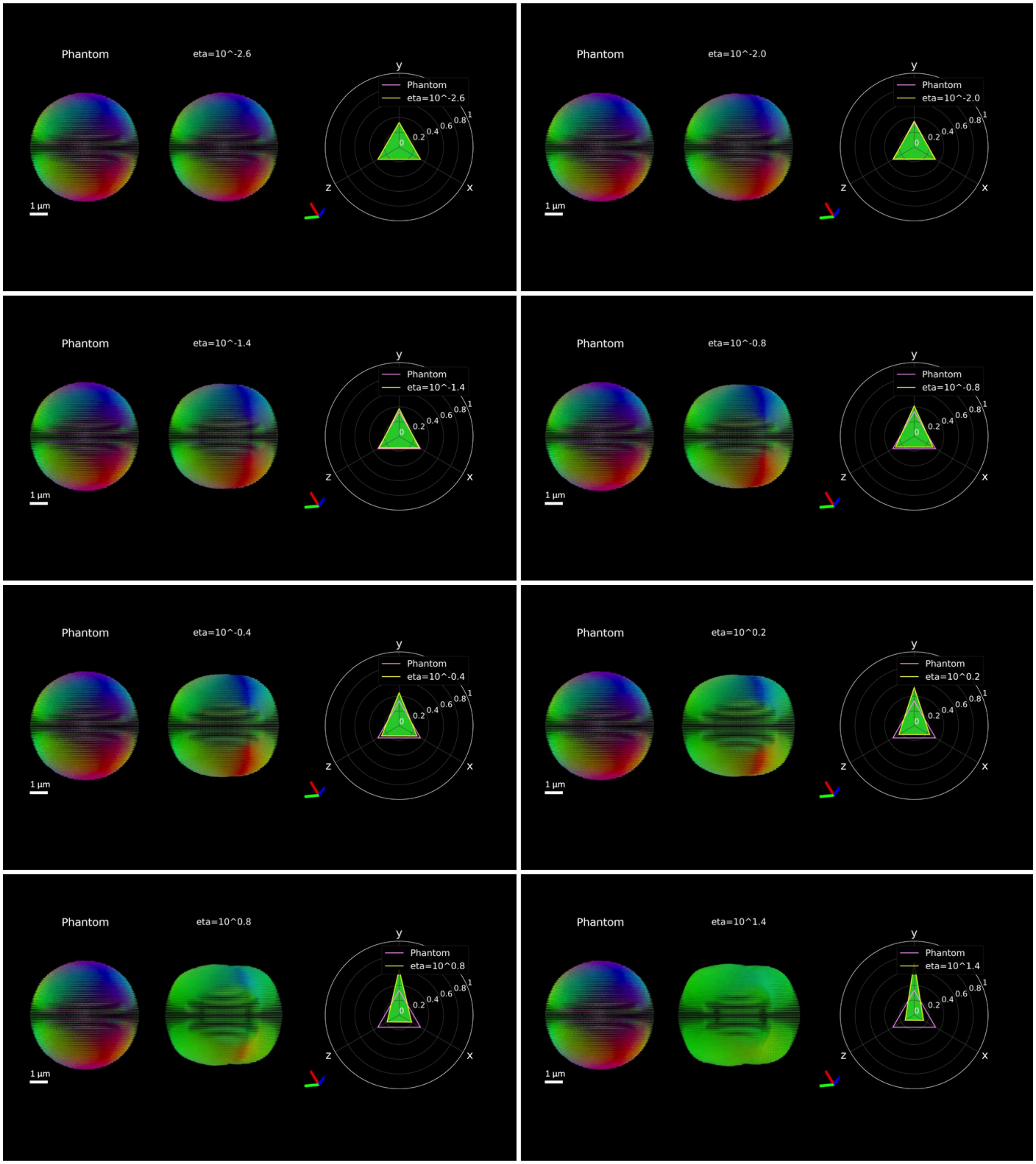

Fig. R3 (montage of **Supplementary Video 4**), **Bias errors caused by regularization parameter in SVD reconstruction on simulated data.** Synthetic shell phantom with molecular orientations perpendicular to the surface (left). SVD reconstructions at different regularization parameters η ('eta') values are visualized in the peak orientations (middle) and the radar chart (right) counts the x, y and z proportion of all peak orientations. Increasing the regularization parameter results in structural distortion and orientations biased towards the y-axis.

6. In Fig 3c, it is not clear why the cell nucleus appears entirely in blue color. The authors do not seem to give a possible explanation (line 275), however it would be good to have one since this is quite puzzling.

RE 18: We thank the reviewer for this careful observation. We have dug into this question and discussed with our biologists – we preliminarily reasoned that the Alexa Fluor 488 phalloidin has stained genuine nuclear F-actin, which is showing a strong polarization signal (GFA = ~0.2, **Supplementary Fig. 11c**). This is consistent with published reports of phalloidin-detectable nuclear F-actin under conditions of mechanical stress and DNA damage [1,2]. Phalloidin is widely regarded as a highly specific probe for F-actin, owing to its low non-specific binding. As far as we learnt, nuclear F-actin signals labelled by phalloidin are typically much weaker than the abundant cytoplasmic F-actin signal, which has made them difficult to distinguish using conventional intensity-based imaging. Here the polarization-resolved analysis segments the phalloidin signal according to fluorophore orientation, allowing weak nuclear F-actin structures to be more readily distinguished from the surrounding cytoplasmic signal. However, we also want to note that it's hard for us to consolidate this observation without additional new data and further exploration, e.g., to fully exclude other possibilities, so we'll post our explanation but be conservative on this point.

[1] Lamm, N. et al. Nuclear F-actin counteracts nuclear deformation and promotes fork repair during replication stress. Nat Cell Biol 22, 1460–1470 (2020).
[2] Belin, B. J., Lee, T. & Mullins, R. D. DNA damage induces nuclear actin filament assembly by Formin-2 and Spire-1/2 that promotes efficient DNA repair. eLife 4, e07735 (2015).

We have included the explanation and revised the text in Lines 284-291 on Page 7:
"The eGRL results revealed differences between the orientation distributions of fluorophores located near the nucleus and those decorating flat or slender filaments. Fluorophores near the nucleus were predominantly oriented along a single direction, whereas those decorating flat or slender filaments exhibited a range of orientations that correlated with the long axes of the actin filaments (**Supplementary Fig. 11**). We reasoned this difference may relate to the distinct alignment[37] between the nuclear F-actin[38,39] architecture and the surrounding cytoplasmic and plasma membrane-associated F-actin cytoskeleton, but consolidating this suspicion may require further experimental validation."

[37] Davidson, P. M. & Cadot, B. Actin on and around the Nucleus. Trends in Cell Biology 31, 211–223 (2021).

[38] Lamm, N. et al. Nuclear F-actin counteracts nuclear deformation and promotes fork repair during replication stress. Nat Cell Biol 22, 1460–1470 (2020).
[39] Belin, B. J., Lee, T. & Mullins, R. D. DNA damage induces nuclear actin filament assembly by Formin-2 and Spire-1/2 that promotes efficient DNA repair. eLife 4, e07735 (2015).

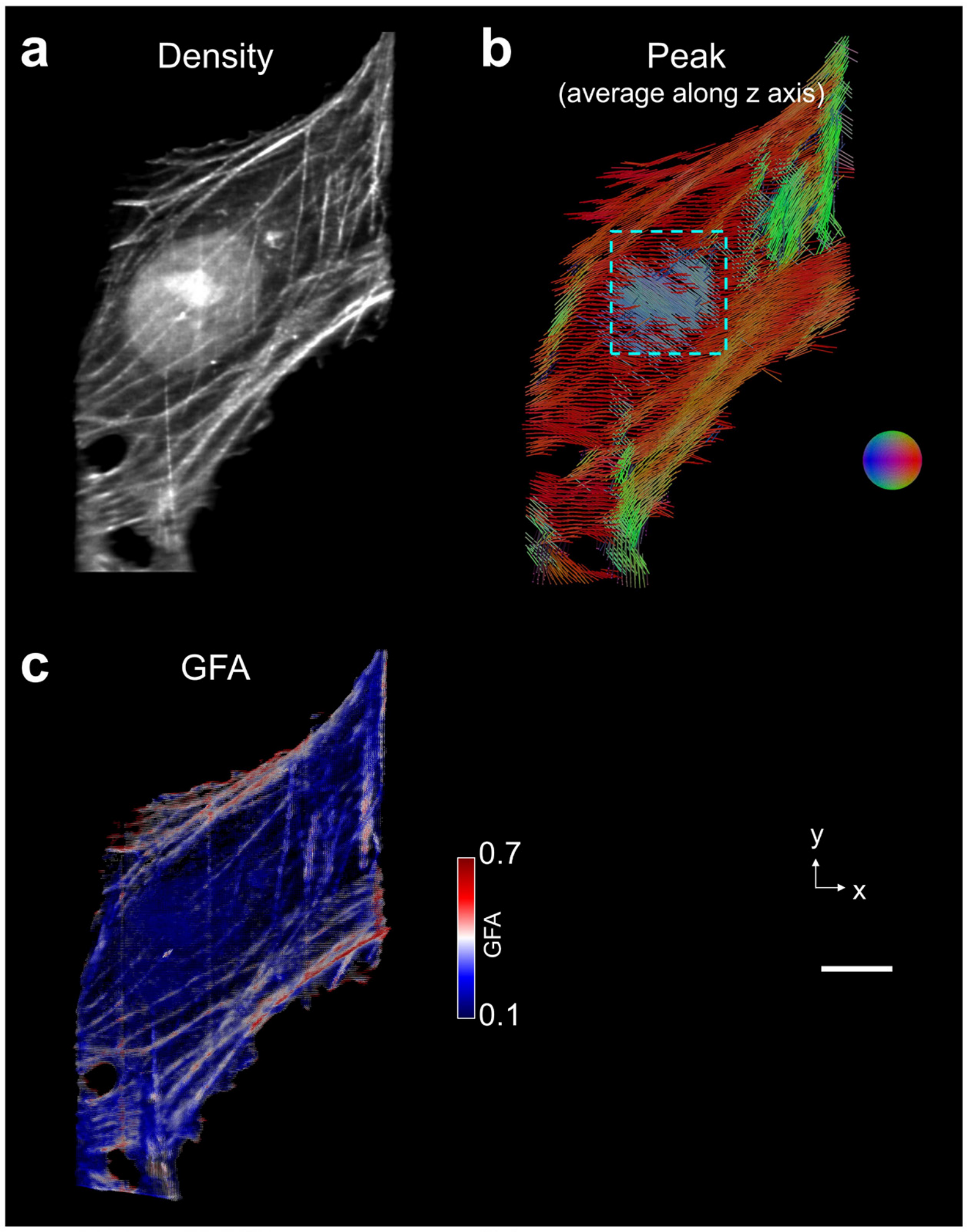

Revised **Supplementary Fig. 11**, **Differential orientation distribution of fluorophores close to nucleus versus elsewhere in U2OS cell. a)** Density map (shown in average-intensity projection) of U2OS cell labeled with Alexa Fluor 488 phalloidin (see also **Fig. 3c**) reconstructed by eGRL. **b)** Orientation map shown in averaged projection. The outlined region highlights distinct orientation distribution near the nucleus compared to surrounding areas. **c) GFA map of the U2OS cell**. Scale bar: 10 µm.

7. In the results shown in Fig 3, it would be good to see, in addition with the orientation information, a clearer quantification of the order parameter (OP) obtained, showing images of OP.

Similarly in Fig. 4 i,j: some profiles of OPs are given but it would be clearer to see an image of it (similarly as in Fig. 4e).

RE 19: We thank the reviewer for this helpful suggestion. We have now added the corresponding quantitative maps in the revised **Fig. 3** and **Fig. 4**. As clarified in **RE 16**, GFA and OP describe different aspects of molecular orientation. GFA measures how concentrated or dispersed the molecular orientations are, without requiring a predefined reference direction. In contrast, OP measures how strongly the molecular orientations are aligned with a selected reference direction.

For the samples shown in **Fig. 3**, there is no obvious predefined reference direction that we aimed to test. We therefore used GFA maps to present the local degree of orientational anisotropy. Specifically, GFA panels for the GUV and xylem samples have been included in **Fig. 3a** left and **Fig. 3f** right, whereas the GFA map of the U2OS sample has been placed in **Supplementary Fig. 11c** due to space limitations.

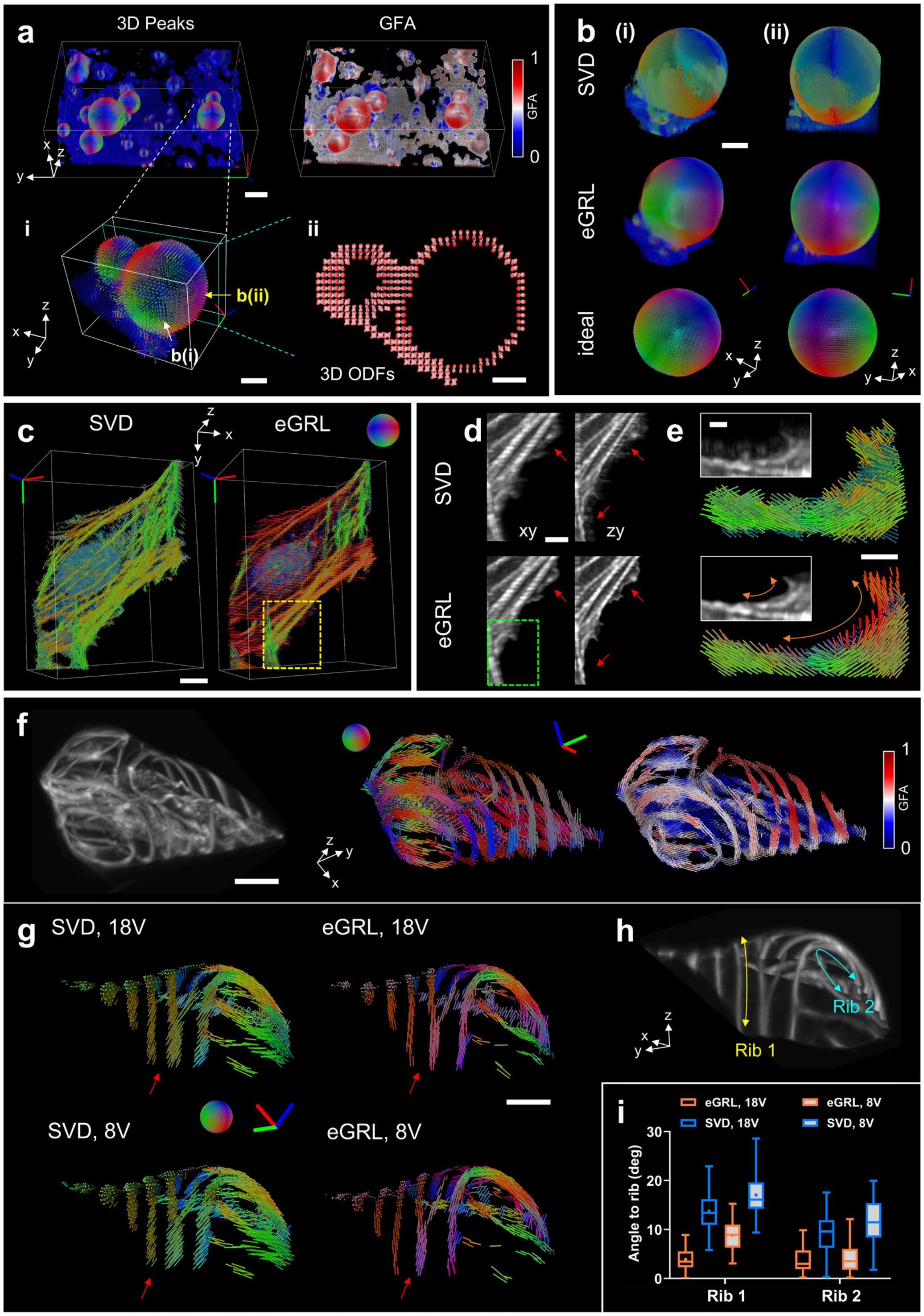

Revised **Fig. 3, Spatio-angular structure reconstructions on multiple fixed samples. a)** eGRL reconstruction of FM1-43 labelled GUVs acquired by pol-diSPIM, shown in peak orientation (left) and GFA (right) map. i, Higher-magnification view of a pair of GUVs (~ 10 µm and ~ 5 µm diameter); ii, ODF map of the slice indicated by the cyan dashed rectangle in i. **b)** The 10 µm GUV viewed from two different perspectives as indicated by the white and yellow arrows in **a**. SVD

reconstruction (top), eGRL reconstruction (middle) and ideal peak orientation maps (bottom, for reference) are presented. Prior knowledge suggests that molecular orientations should be normal to the membrane surface, which is better approximated by the eGRL result than the SVD reconstruction. **c)** Reconstructions by SVD and eGRL of a fixed U2OS cell labeled with Alexa Fluor 488 phalloidin. **d)** The lateral (left) and axial (right) maximum-intensity projections from the density map of the yellow dashed rectangular region in **c**. Note eGRL produces a better resolved density map with fewer artifacts (red arrows). **e)** Higher magnification views of the density and peak orientation maps of the green dashed rectangular region in **d**. The orange curved bidirectional arrows indicate that the peak orientations reconstructed by eGRL smoothly change along the tangential direction, while those by SVD are disorganized. **f)** The density (left), peak orientation (middle) and GFA (right) maps of a xylem cell (labelled by Pontamine fast scarlet) reconstructed by eGRL. **g)** Comparison of the peak orientations reconstructed with 18 and 8 polarization modulations by eGRL and SVD, showing that eGRL better preserves reconstruction than SVD even when reducing the number of polarizations from 18 to 8 by eGRL. Note the bottom of the cell has been cut away for easier visualization. **h)** Density perspective to accompany **g**. **i)** The angles of the peak orientations deviating from the rib directions at ~260 points uniformly selected on each rib (yellow and cyan bidirectional arrows) in **g**. Scale bars: **a** top 10 µm, **a** i-ii 5 µm, **b** 5 µm, **c** 10 µm, **d** 5 µm, **e** 2 µm, **f-h** 10 µm.

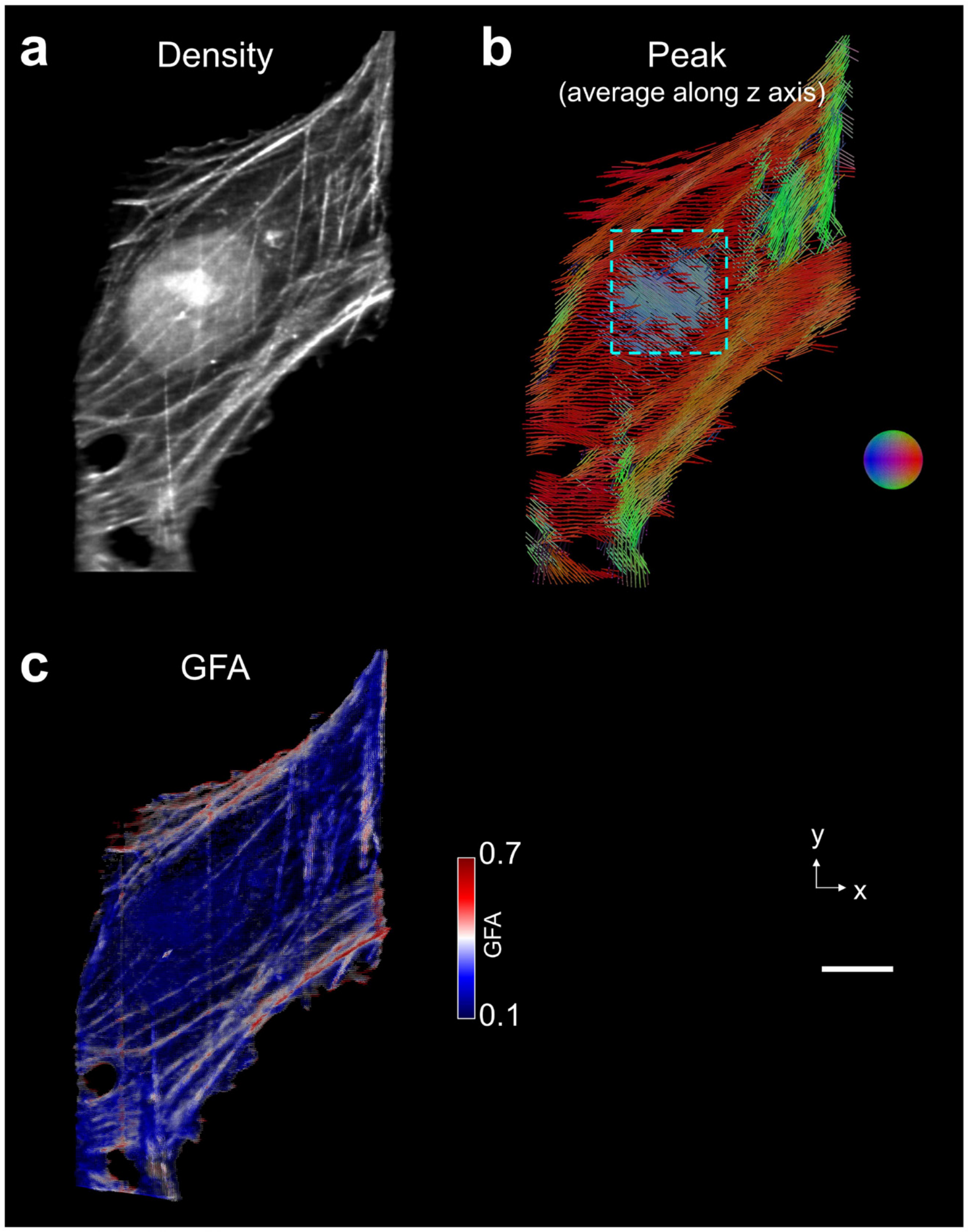

Revised **Supplementary Fig. 11**, **Differential orientation distribution of fluorophores close to nucleus versus elsewhere in U2OS cell. a)** Density map (shown in average-intensity projection) of U2OS cell labeled with Alexa Fluor 488 phalloidin (see also **Fig. 3c**) reconstructed by eGRL. **b)** Orientation map shown in averaged projection. The outlined region highlights distinct orientation distribution near the nucleus compared to surrounding areas. c) GFA map of the U2OS cell. Scale bar: 10 µm.

For the samples in **Fig. 4**, the nanowire direction provides a clear reference axis. We therefore used OP maps to quantify how strongly the molecular orientations align with the nanowire direction. We have included the OP map along two reference directions in **Fig. 4i** top and **Fig. 4j** top.

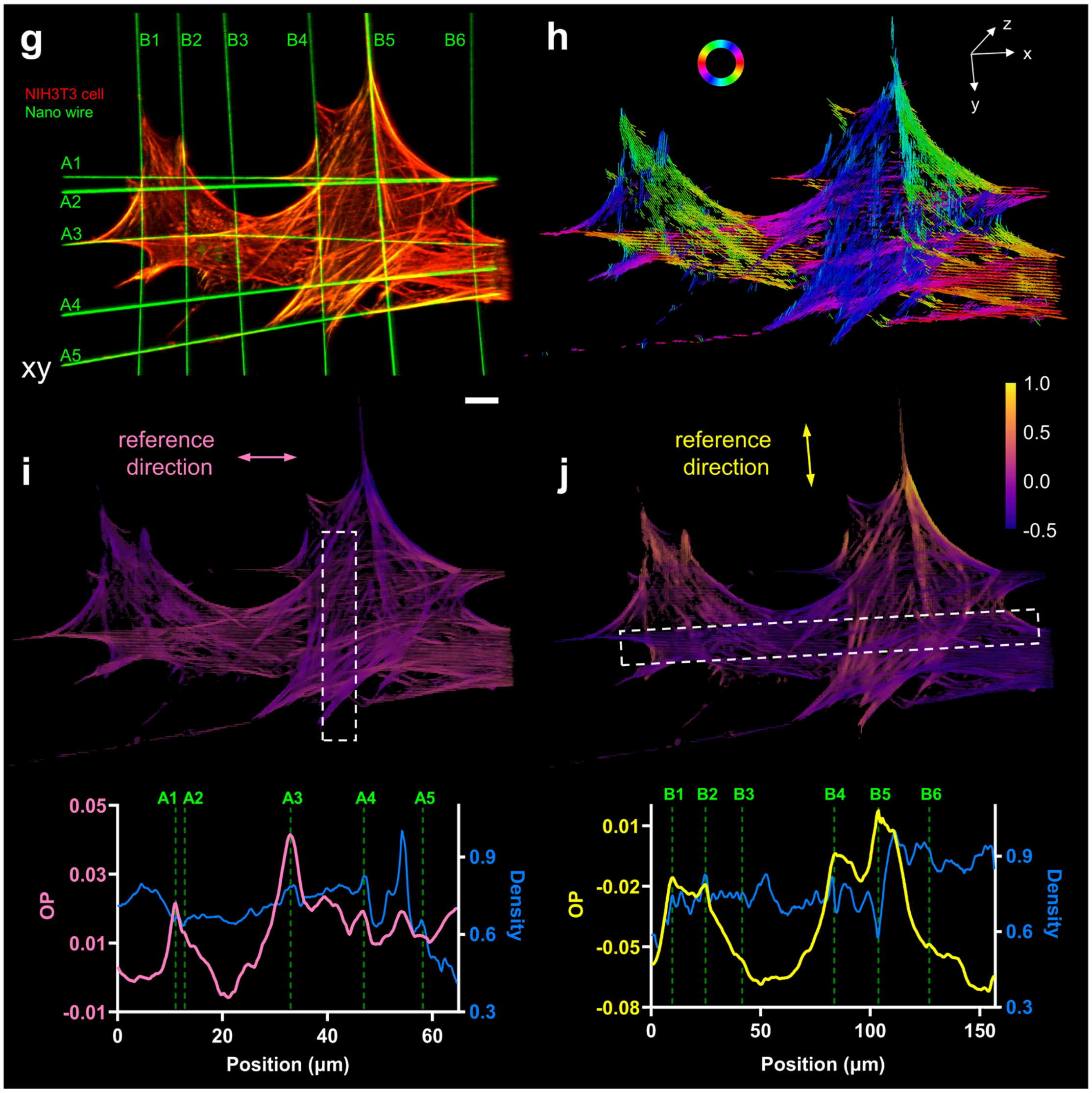

Revised **Fig. 4g-j**, **Revealing correlations between nanowire directions and the molecular orientation of actin. g)** Density maximum intensity projections showing another fixed NIH3T3 cell (in red color) grown on crosshatched nanowires (in green color) as for **a**. All the nanowires are numbered based on the approximate direction (A for horizontal wires, B for vertical ones). **h)** The peak orientation map of the actin in **g** reconstructed by eGRL, with a different colorbar/colormap encoded in 2D XY plane due to the narrow thickness of cell. **i)** Top, OP map along the reference direction defined as the bidirectional pink arrow. Bottom, OP profile along the long side of the white dashed rectangle in **i** top (averaged in the short side and z axis). The OP profile passes through wire A1-A5 and the corresponding wire positions are marked on the profile. Note the wires are mostly coincident with the local maxima on the OP curve. **j)** Top, OP map along the reference direction defined as the bidirectional yellow arrow. Bottom, OP profile along the long side of the white dashed rectangle in j top (averaged in the short side and z axis). The OP profile

passes through wire B1-B6. As in **i**, the wires often coincide with the local maxima on the OP curve. Scale bars: **g** 10 µm.

8. In Line 344, Ref 47 is cited to describe effect of actin alignment in cells, however this does not seem to be the most appropriate illsutration as this paper is rather focussed on the extra cellular matrix alignment. The observed loss of OP could be rather due to the occurrence of a mixture of actin filaments populations, both from the stress fiber bundles and a more isotropic surrounding. Can the authors comment on this possible mixture of populations?

RE 20: We appreciate the reviewer's insightful comment and agree that a more conservative interpretation is appropriate. In the initial manuscript, we raised two possible explanations: "nanowire promotes actin accumulation and growth in adjacent regions along the nanowire direction" and "the rise in local stress due to the cellular elongation along nanowires".

We consider the first explanation to be the most plausible, and it is also consistent with the reviewer's perspective. Regions closer to the nanowires show stronger preferential alignment from stress fiber bundles, whereas regions farther from the nanowires contain a more mixed population of actin filaments with more isotropic ODFs, resulting in reduced OP. We have therefore revised the text to make this interpretation more explicit.

The second explanation, regarding a possible increase in local stress, was intended to describe relatively higher local stress due to more actin-fiber accumulation. However, we agree that this wording and citation of Ref 47 may be misleading, as it could imply that pre-existing actin fibers experience increased stress after nanowire-induced elongation. Such a conclusion would require direct evidence, such as tracking the OP of the same actin filament before and after treatment or performing independent mechanical measurements. To avoid overinterpretation, we have removed the statement related to stress induction.

We now have revised the text to focus on the more conservative conclusion that stress fiber actin bundles preferentially accumulate and align near the nanowires, in Lines 355-359 on Page 9:
"The fluorescent probes bound to the cytoskeleton firmly anchor along the direction of actin filaments, indicating that the accumulation and growth of stress-fiber-like actin bundles in adjacent regions along the nanowire direction. In contrast, regions farther from the nanowires likely contain a more mixed population of actin filaments with more isotropic orientation distribution."

9. In Lines 436 to 459, the section addressing cell membranes labelled with FM1-43 shows GFA maps and not anymore OP maps, is there a reason for this and why not use the same quantity to quantify such order parameter? Also there is no clear explanation given for the effect of GFA changes close to the coverslip.

RE 21: We thank the reviewer for their feedback. We would like to clarify that, GFA and OP describe different aspects of molecular orientation (overall anisotropy vs. preference in one interested direction). Please see our detailed response in **RE 16** and **RE 19** above.

Membrane fluidity has been shown to be reduced in confined geometries and in apposition to stiff surfaces (e.g., glass), as has been previously observed with in vitro reconstitution systems [1]. Previous SMOLM studies also suggest that dye wobble is related to the degree of intermolecular spacing and fluidity within the membrane [2–4]. We therefore speculate that the rigidity of the coverslip may reduce the local membrane fluidity, thereby constraining the orientational spread of FM1-43 molecules and leading to higher GFA values near the coverslip. This is also supported by single-molecule imaging observations reported in Ref. [5].

[1] Matsuzaki, T. et al. Simultaneous visualization of membrane fluidity and morphology defines adhesion signatures of cancer cells. Proc. Natl. Acad. Sci. 121, e2412914121 (2024).
[2] Lu, J. et al. Single molecule 3D orientation imaging reveals nanoscale compositional heterogeneity in lipid membranes. Angew. Chem. Int. Ed. 59, 17572–17579 (2020).
[3] Ding, T. & Lew, M. D. Single-molecule localization microscopy of 3D orientation and anisotropic wobble using a polarized vortex point spread function. J. Phys. Chem. B 125, 12718–12729 (2021).
[4] Wu, T., Lu, J. & Lew, M. D. Dipole-spread-function engineering for simultaneously measuring the 3D orientations and 3D positions of fluorescent molecules. Optica 9, 505–511 (2022).
[5] Zhang, O. et al. Six-dimensional single-molecule imaging with isotropic resolution using a multi-view reflector microscope. Nat. Photon. 17, 179–186 (2023).

We have now added the explanation and cited the relevant papers in the revised manuscript, Lines 470-474 on Page 11:
"We found that membranes displayed higher GFA near the coverslip than elsewhere on the cell (**Fig. 5h**, see also polarization-insensitive negative control in **Supplementary Fig. 21d**, excluding motion as a cause of differential GFA), consistent with previous reports that the rigidity of the coverslip reduces the membrane fluidity[62] and thus constrains the spread distribution of FM1-43 molecules[2,63]."

[2] Zhang, O. et al. Six-dimensional single-molecule imaging with isotropic resolution using a multi-view reflector microscope. Nat. Photon. 17, 179–186 (2023).

[62] Matsuzaki, T. et al. Simultaneous visualization of membrane fluidity and morphology defines adhesion signatures of cancer cells. Proc. Natl. Acad. Sci. 121, e2412914121 (2024).
[63] Ding, T. & Lew, M. D. Single-Molecule Localization Microscopy of 3D Orientation and Anisotropic Wobble Using a Polarized Vortex Point Spread Function. J. Phys. Chem. B 125, 12718–12729 (2021).

10. In Fig. 5, it would be nice to also display GFA maps, as the degree of order is as important as orientation.

RE 22: We agree with the reviewer that anisotropy information is important in addition to orientation. Following the reviewer's suggestion, we have added GFA maps for the HeLa cells and macrophages in revised **Supplementary Figs. 18a** and **21b**, and included the GFA values of the U2OS samples in the histogram panel in **Fig. 5**.

Due to space limitations, we placed the HeLa and macrophage GFA maps in the Supplementary Information rather than the main figure. However, we would be happy to further adjust the layout and include the GFA panels in the main text if the reviewer feels that any existing panels in the main figure are less essential or could be replaced.

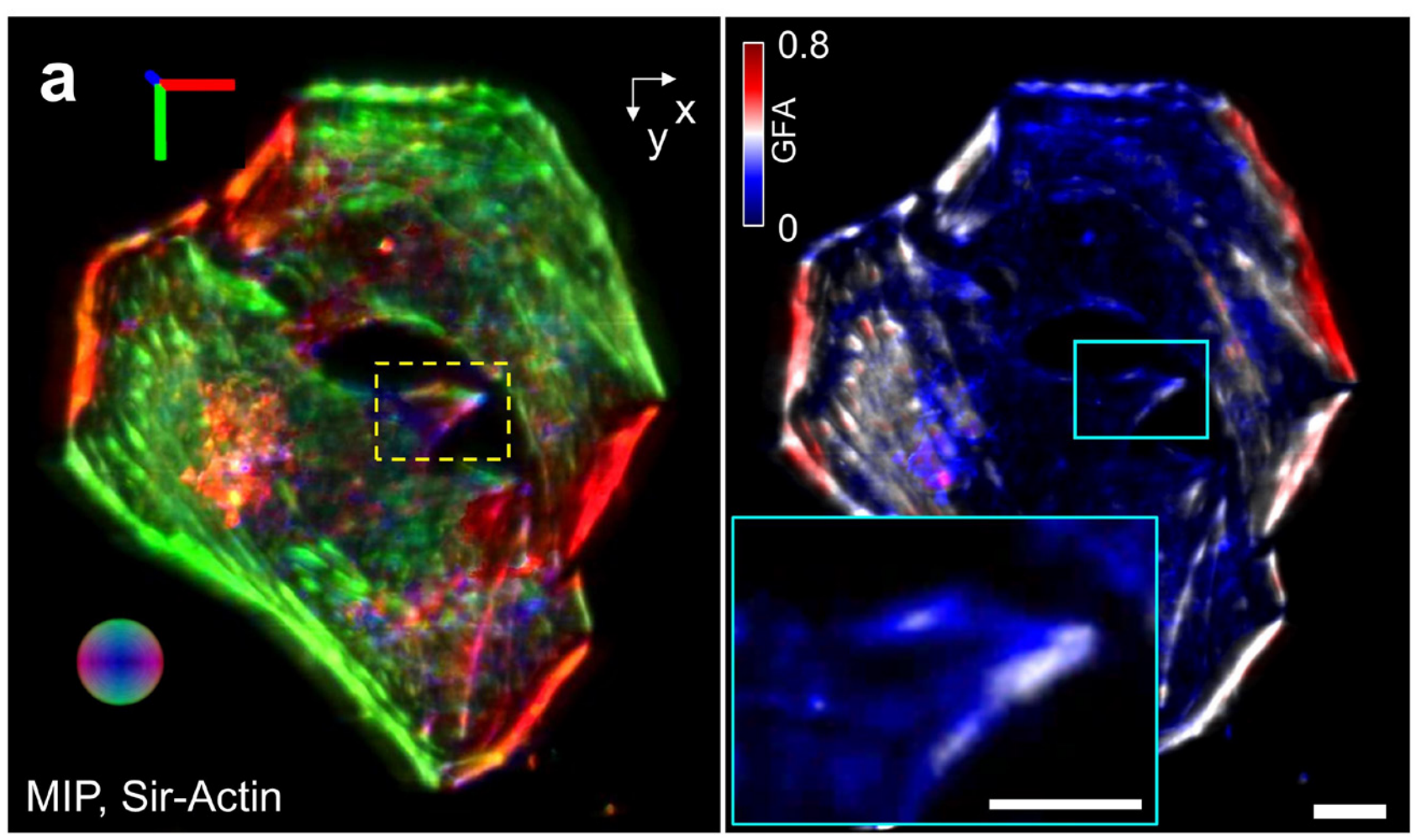

Revised **Supplementary Fig. 18a**, **Orientational distribution and dynamics on live HeLa cell protrusion. a)** The principal orientation map and GFA map of a live HeLa cell labelled with Sir-Actin and excited at 640 nm. The inset shows relatively high GFA values (0.2~0.4) on the edge of protrusions compared with surrounding regions in protrusion. Scale bar: **a** 5 μm.

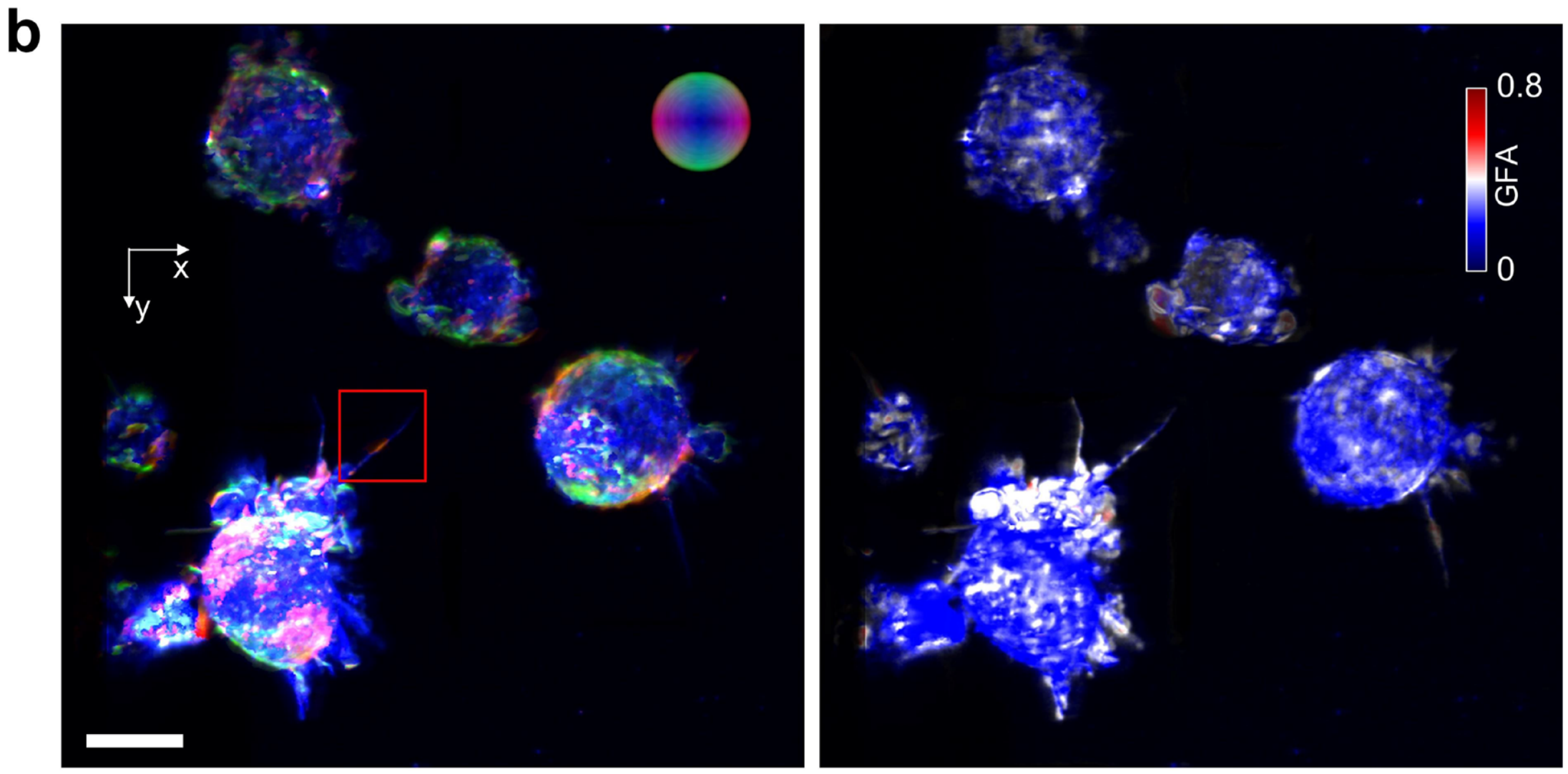

Revised **Supplementary Fig. 21b**, **Excluding motion artifacts from analysis of macrophage membrane protrusions. b)** Maximum intensity projection of multiple FM1-43 labelled live macrophage cells colored by principal orientations (left) and GFA values (right) at each voxel. Scale bars: **b** 10 µm.

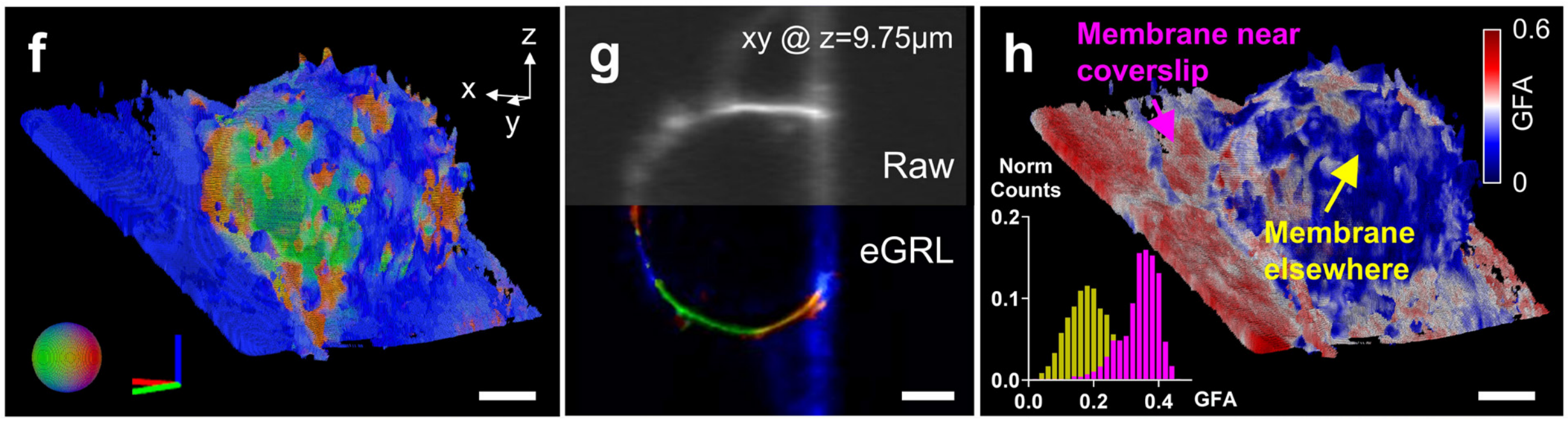

Revised **Fig. 5f-h**, **Observing spatio-angular dynamics in living cells. f)** The principal orientation map of a live U2OS cell labeled with FM1-43. **g)** Comparison of raw data and the eGRL reconstruction colored by the principal orientation, in the lateral slice at z depth of 9.75 µm. **h)** GFA map and histogram of GFA values at the initial time point, showing the higher GFA in the vicinity of the coverslip surface (coated with poly-L-Lysine) than elsewhere on the cell membrane, likely because the former constrains the membrane surface more than elsewhere in the cell. Scale bars: **f-i** 5 µm.

11. Fig. 5iii shows an interesting twisting behavior: how is this observation reproducible? it would be good to know if this has been observed on several instances or just this specific cell. Can this observation be related to the dynamic behavior of actin at the edge of the cell, involving nucleation/capping and polymerization mechanisms?

RE 23: We are pleased that the reviewer found this observation interesting. Another example of twisting is shown in **Supplementary Fig. 19d** i (see **Fig. R4** for a magnified view). As indicated by the arrow in **Fig. R4d**, the orientation along the protrusion gradually transitions from in-plane (within the xy plane, approximately along the y-axis) to out-of-plane (perpendicular to the xy plane, approximately along the z-axis).

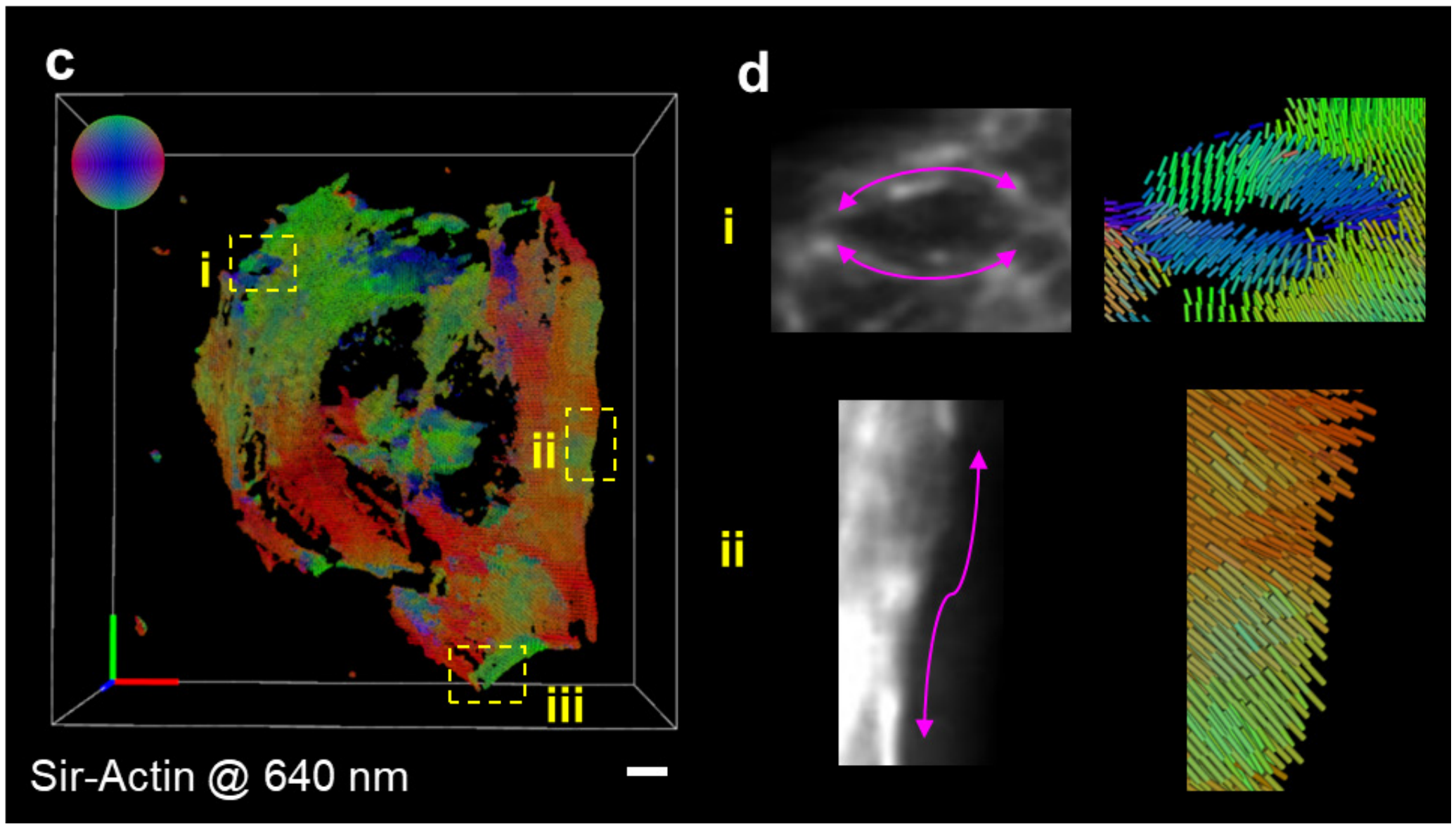

Supplementary Fig. 19c, **d**, **Comparison of the overall orientation distribution pattern of actin filaments in fixed and live HeLa cells labelled with different dyes. c)** A live HeLa cell labeled with Sir-Actin and imaged under 640 nm excitation. **d)** Higher-magnification views of the orientation map corresponding to the dashed rectangle regions in **c**, illustrating predominantly perpendicular orientations of the Sir-Actin label relative to actin filaments (profiles shown by magenta arrows), in stark contrast to the images in **a**. Additionally, representative ODF maps corresponding to the cyan dashed regions are shown for an in-depth comparison. **Scale bars**: **c** 5 µm, **d** 1 µm.

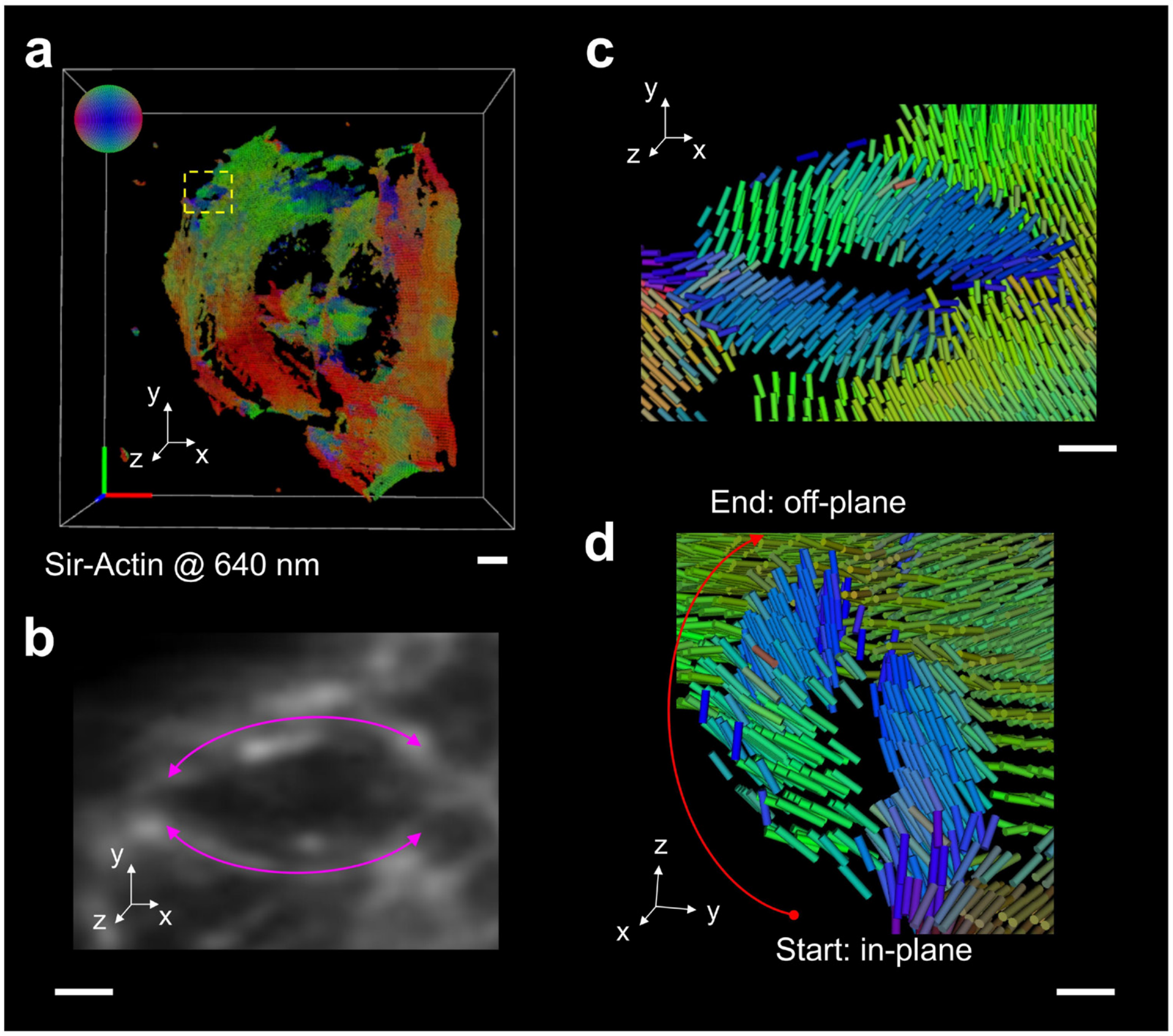

Fig. R4, Magnified views to the twisting structure of the HeLa cell in Supplementary Fig. 19d. a) Overall view of the HeLa cell, shown in peak orientation map. **b**, **c)** Higher-magnification views of the intensity **b** and orientation **c** map corresponding to the dashed rectangle regions in **a**. **d)** An additional magnified view of the same region as **b** and **c**. The arrow highlights a gradual twisting along the protrusion, transitioning from in-plane to out-of-plane orientation. Scale bars: **a** 5 µm, **b-d** 1 µm.

However, the extended and more pronounced twisting observed in **Fig. 5b** iii was only identified in one HeLa sample among the six samples we imaged. To further support this observation, we have revised **Supplementary Fig. 18**, where:

1) the twisting pattern is consistently observed across all time points (as already shown), which reduces the likelihood that the observation arises from random noise or transient fluctuations.

2) we newly include GFA analysis demonstrating that the edges of the protrusions (i.e., the twisting regions) exhibit relatively higher GFA values (**Supplementary Fig. 18a** right, inset outlined by cyan, approximately 0.2–0.4) compared to the surrounding background and other regions within the protrusions, indicating a relatively strong orientational anisotropy.

Together, these results strengthen the reliability of the observed twisting behaviour.

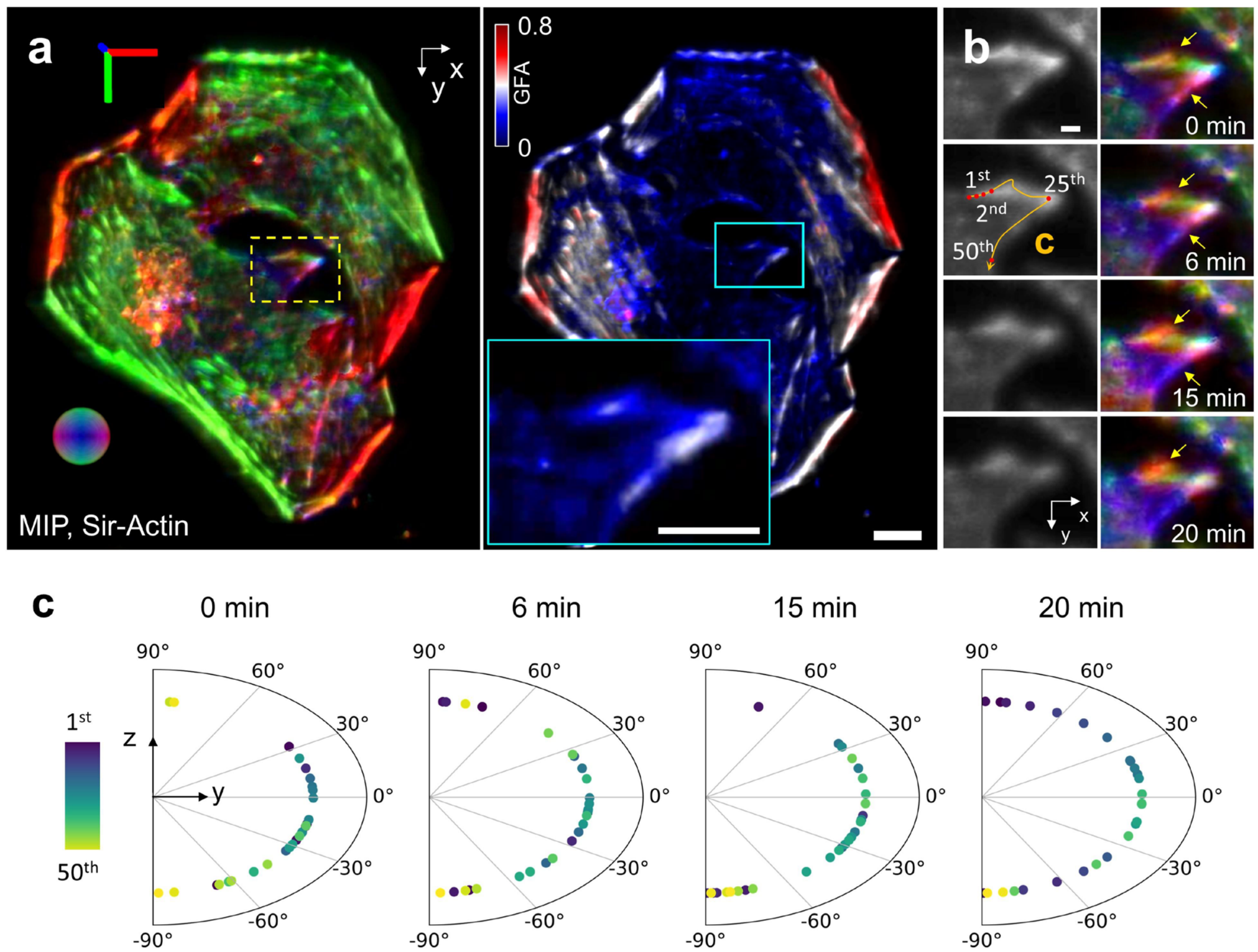

Revised **Supplementary Fig. 18, Orientational distribution and dynamics on live HeLa cell protrusion. a)** The principal orientation map and GFA map of a live HeLa cell labelled with Sir-Actin and excited at 640 nm. The inset shows relatively high GFA values (0.2~0.4) on the edge of protrusions compared with surrounding regions in protrusion. **b)** A dynamic sequence of the same region outlined by the yellow dashed rectangle in **a**. The arrows point out small changes that are hard to observe in the density map but are noticeable in the orientation distribution. **c)** How principal orientation distributes along the actin filament of protrusion. 50 characteristic points were selected uniformly along the trail in **b**. The polar plots are shown for the inclination angle of the principal orientation in the zy plane, with pseudo color indicating the points' order (1st ~ 50th) along the trail. Scale bars: **a** 5 µm, **b** 2 µm.

Regarding the explanation of the mechanisms:

1) our understanding is that, protrusions may often have high levels fascin, especially in cancer cells [1] like the one shown in **Supplementary Fig. 18a** above. Because fascin binds parallel actin filaments with a helical pitch, it can induce rotational torsion resulting in the twisted orientation [2] of filaments shown here. We think that our method provides an excellent tool to study such architectures in live cells, that previously required ultrastructure in fixed cells to uncover.

2) Regarding the potential mechanisms mentioned by the reviewer, we have done some research on literature but found that we could not make a solid one without additional experimental support. Unfortunately, we currently have moved our lab from the institute where the pol-diSPIM instrument was installed and no longer have access to operate the system for more data acquisition. While we do agree it would be good to explore biology applications, we also feel that this manuscript is mostly focused on imaging technique development, and further biological analysis is outside the scope of this manuscript.

[1] Machesky, L. M., & Li, A. Fascin: Invasive filopodia promoting metastasis. Communicative & Integrative Biology, 3(3), 263–270 (2010).
[2] Rajan, S. et al. Actin Bundles Dynamics and Architecture. Biomolecules, 13(3):450 (2023).

12. In Line 703, the calibration procedure seems to be only a recentering of phases of known polarization curves, however distorsions such as ellipticity frequently occur in polarized microscopy. How is this measured and compensated for?

RE 24: We thank the reviewer for raising this important point. In the current implementation, we did not explicitly model or correct for higher-order distortions such as ellipticity.

During calibration we carefully characterized the polarization state at the object plane. In particular, we ensured a high degree of linear polarization with an extinction ratio exceeding 100:1. This indicates that residual ellipticity is relatively small under our experimental conditions. As a result, we expect its impact on the orientation estimation to be limited compared to other sources of uncertainty.

13. Despite the high quality of the results obtained, the examples shown are very close to the previously published work by the authors on the same technique and they do not entirely tackle the practical context of light sheet microscopy. Could the authors show the added value of the method by addressing dynamic tissue samples generally measured with this method (ex. drosophila or c.elegans)?

RE 25: We agree with the reviewer that light sheet microscopy has the powerful capacity to capture dynamics of large samples such as tissues and small animals, and we have the same feeling that it would greatly broaden the impact of our method with demonstration on these samples. But as mentioned above in **RE 23,** we have moved our lab and have no access to the original pol-diSPIM system. So it might be hard for us to collect more data before we have set up a system at our new lab, which is not yet complete due to various upgrades we are making.

However, we'd also like to discuss a few considerations and potential challenges in imaging large samples:
1) Potential optical aberrations in large, live samples. Aberrations typically increase as a function of depth, distorting the system PSF and reducing image quality. While perhaps not a big problem for methods that don't need quantitative analysis, they have the potential to severely distort the methods we describe here. We have now also added a few sentences about it to the **Discussion** of the manuscript, see Lines 510-513 on Page 12:
"Our demonstrations in living cells were limited to relatively slow dynamics due to the need to capture multiple polarization modulations for full 3D polarized fluorescence imaging. …… Potential aberrations should also be considered especially when imaging large specimens. While aberrations may be tolerable for conventional intensity imaging systems, they might be more influential to polarization imaging where quantitative analyses require more accurate and comprehensive modelling of the imaging system."

2) Current experimental speed bottlenecks. Due to the need for additional polarization modulations, the pol-diSPIM system is 3x slower than a conventional light sheet system. While not a limitation for fixed samples but for large-scale dynamic samples, we see a need for higher speed. We had discussed this point in the **Discussion** of the manuscript, see Lines 492-510 on Page 12.

Thus, although we intentionally focused on imaging small samples such as living cells (depth < 20 um) in our original manuscript, we plan to work on larger, tissue-scale specimens in the future.

Other more minor comments:
14. A lot of images in the supplementary figures (ex. Suppl Fig S15,16) do not have axes which permit to known which direction is used to show the sample. It makes it confusing to known which view angle they represent.

RE 26: We thank the reviewer for pointing out these omissions which now have been added.

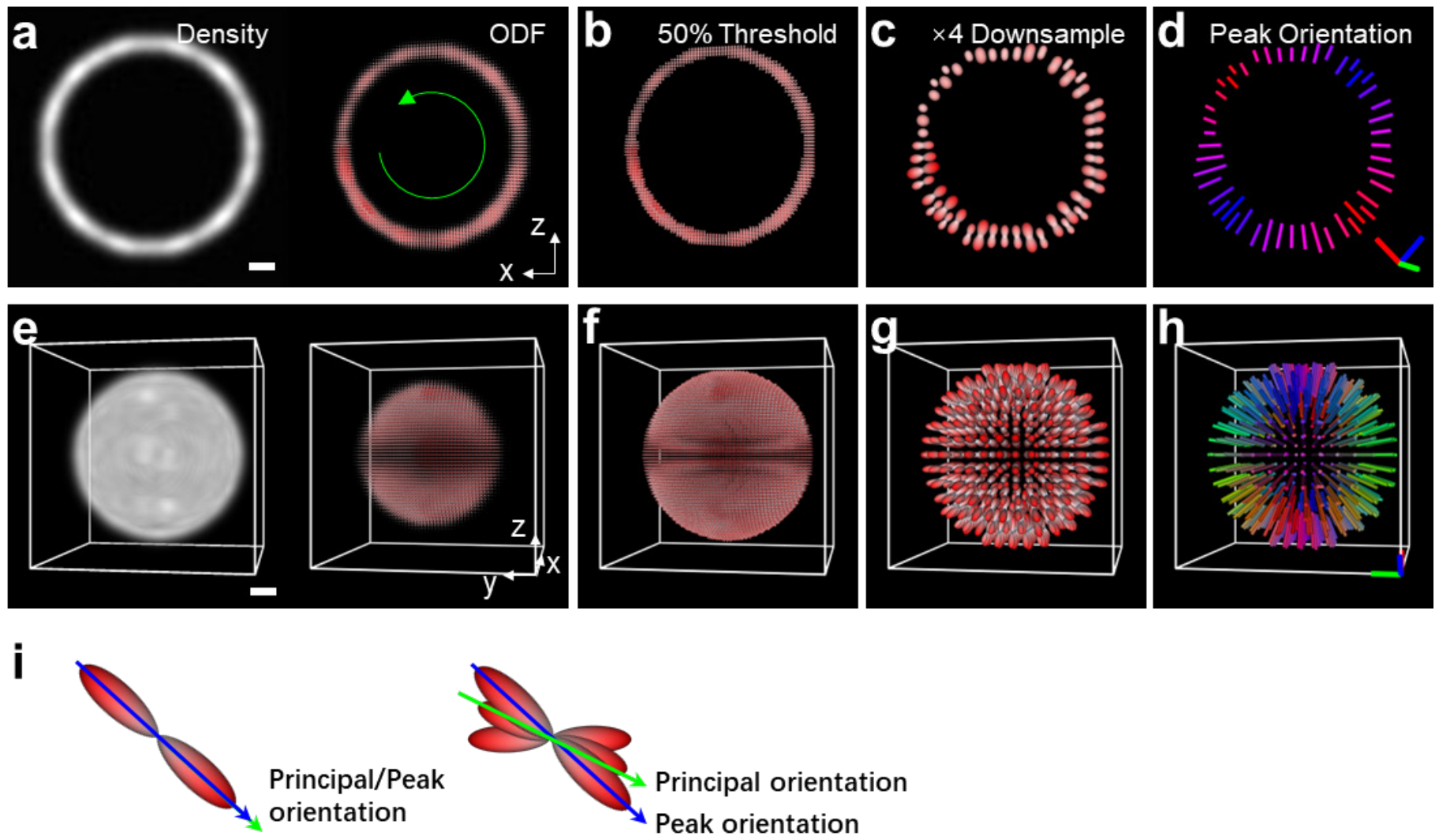

Revised **Supplementary Fig. 1**.

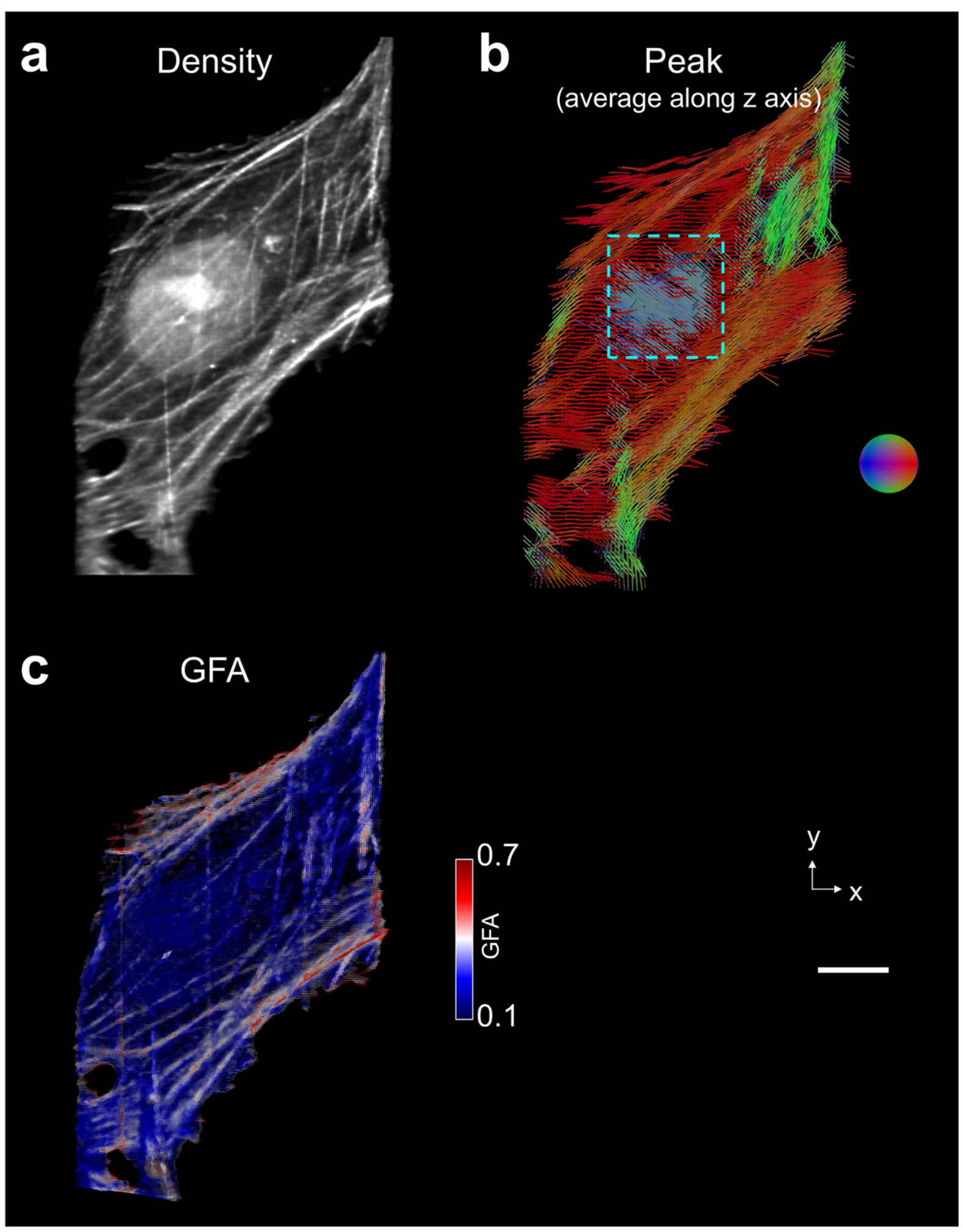

Revised **Supplementary Fig. 11**.

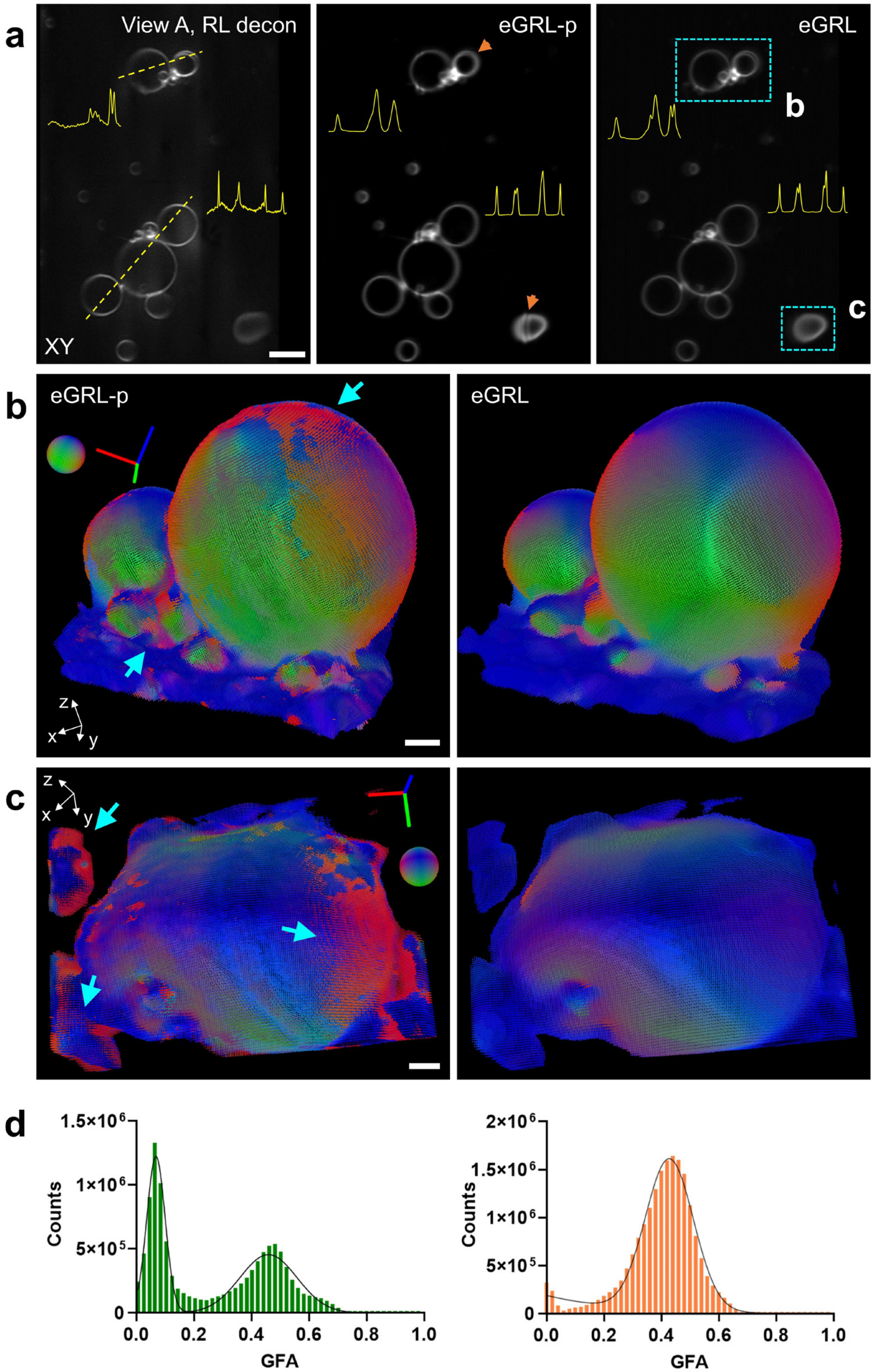

Revised **Supplementary Fig. 12b, c**.

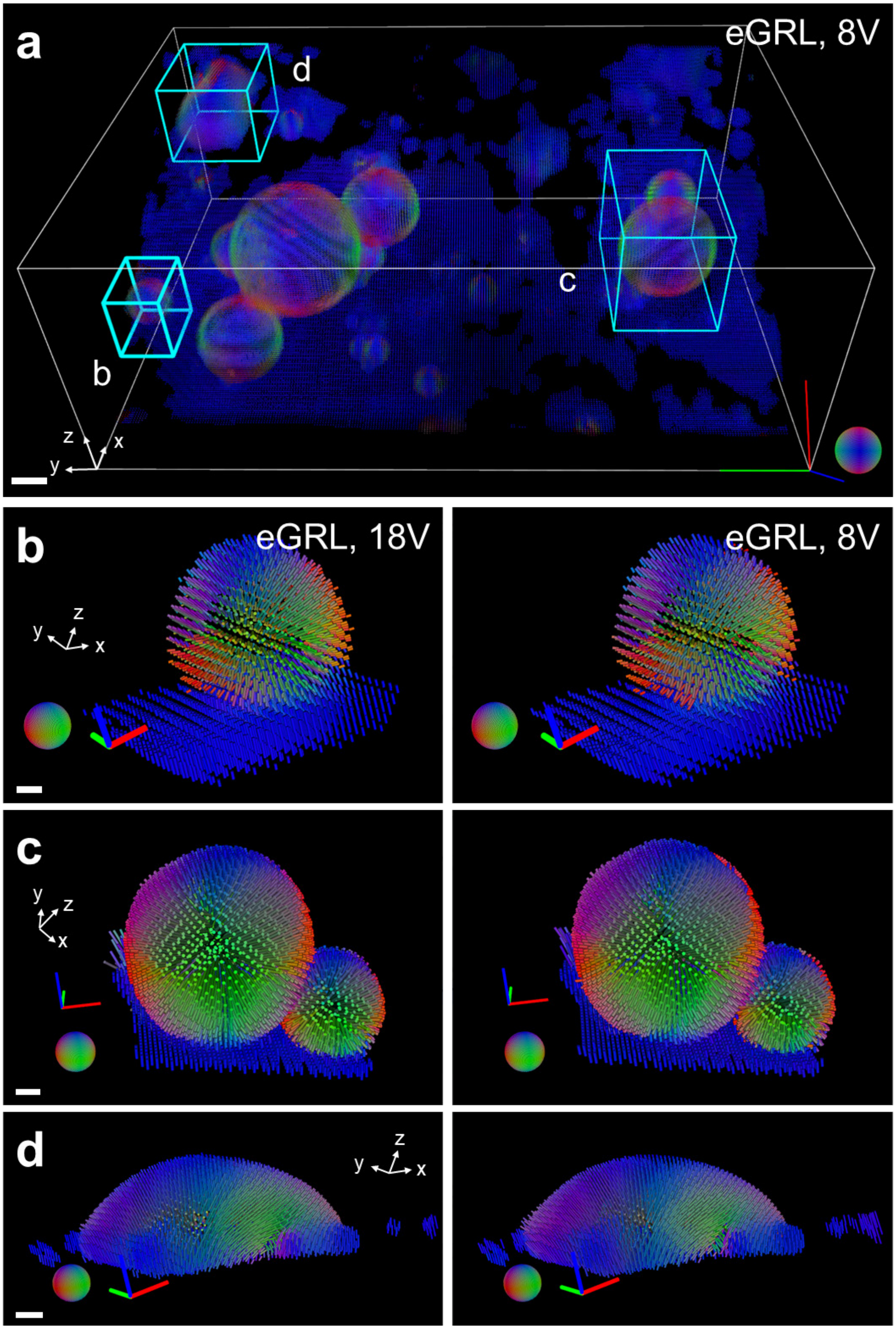

Revised **Supplementary Fig. 13**.

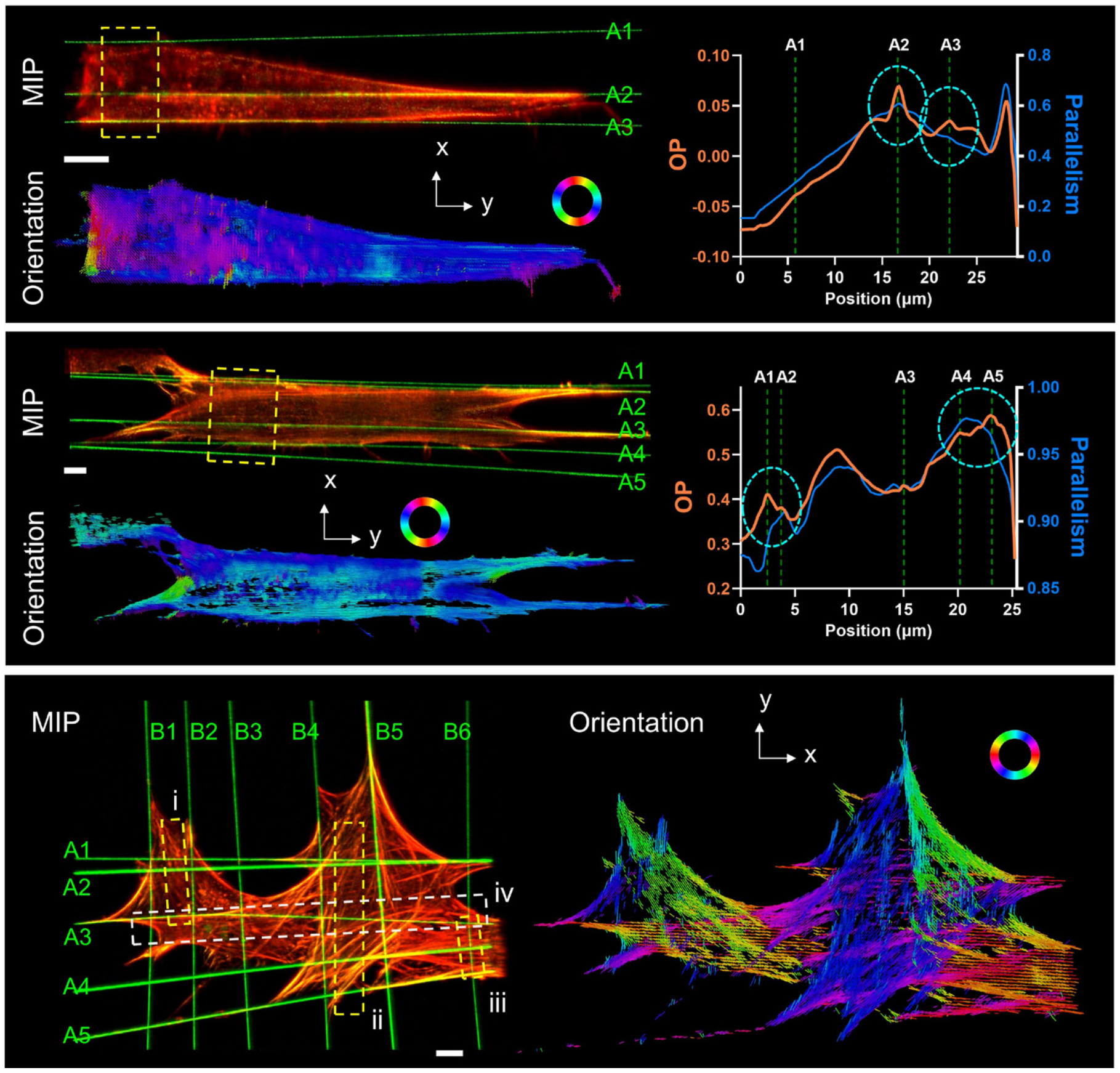

Revised **Supplementary Fig. 16a-c**.

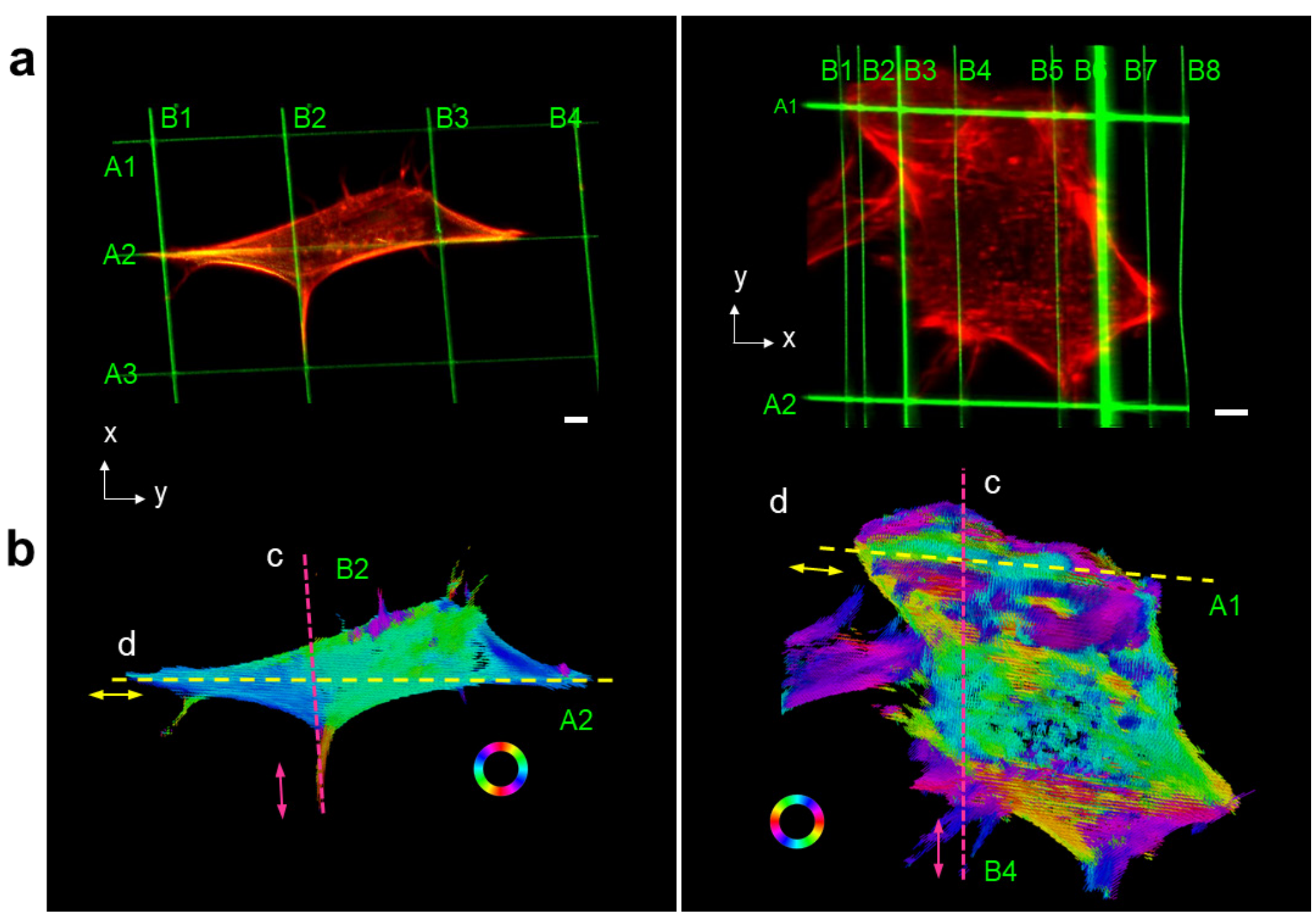

Revised **Supplementary Fig. 17a, b**.

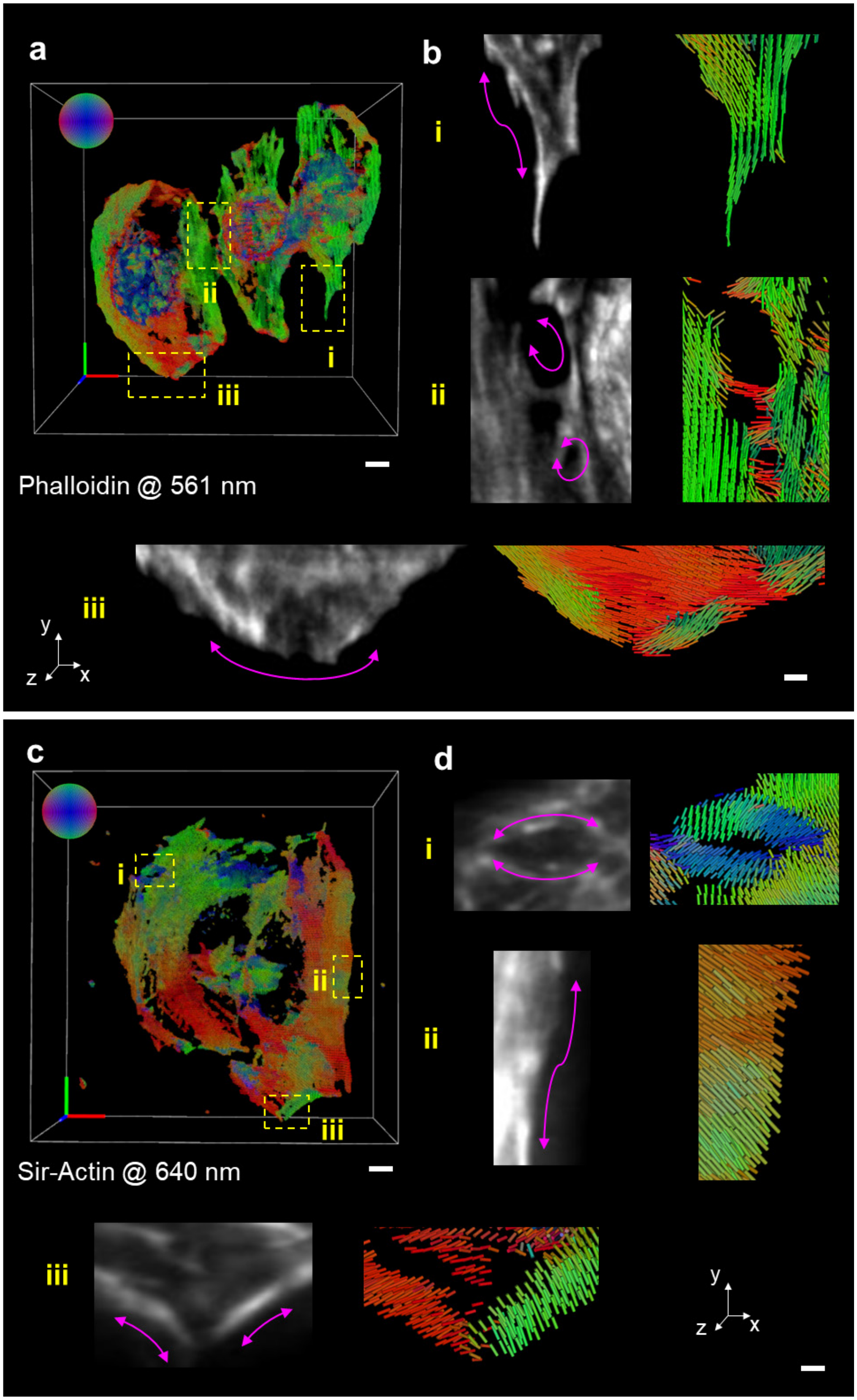

Revised **Supplementary Fig. 19**.

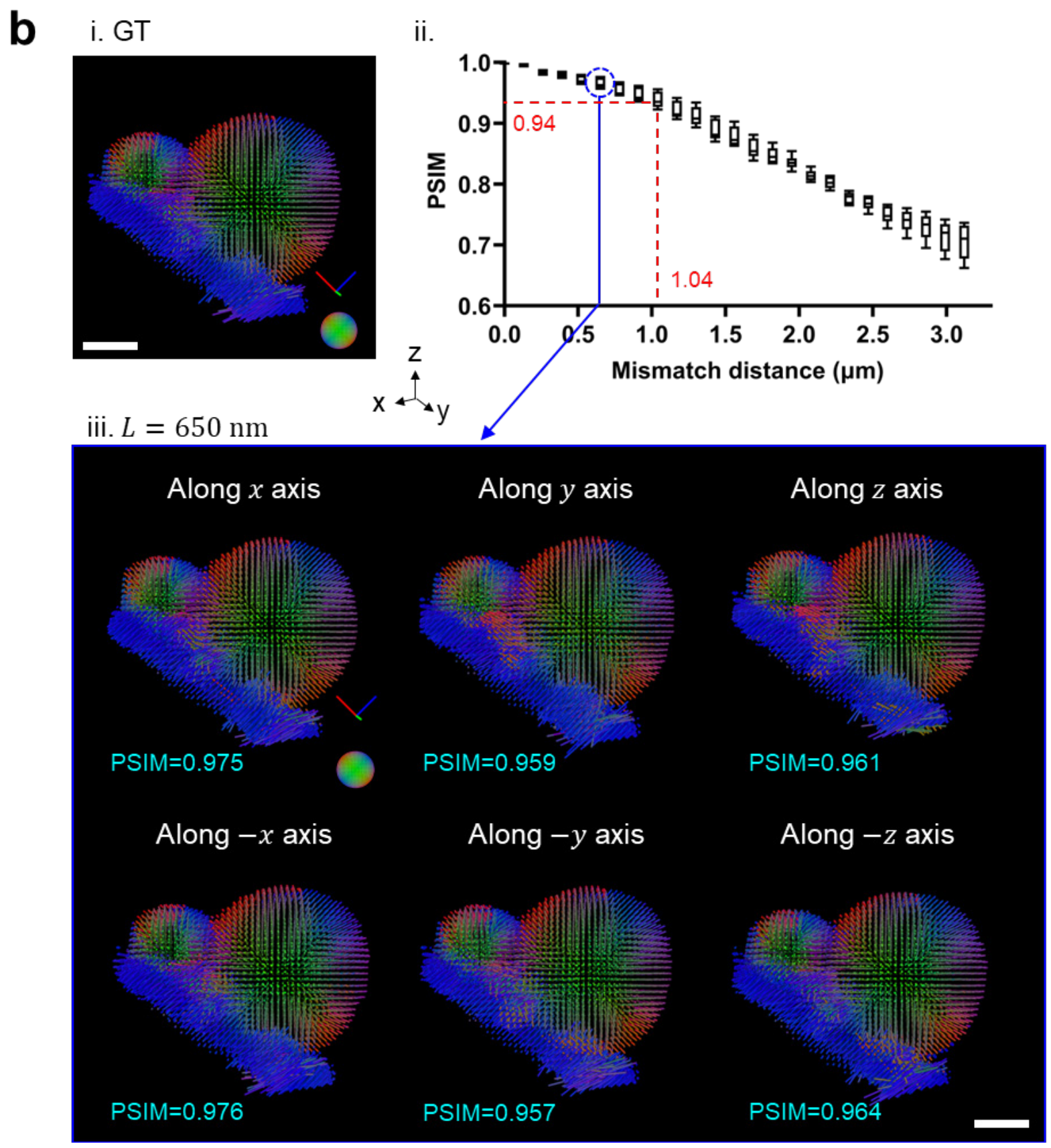

Revised **Supplementary Fig. 23**.

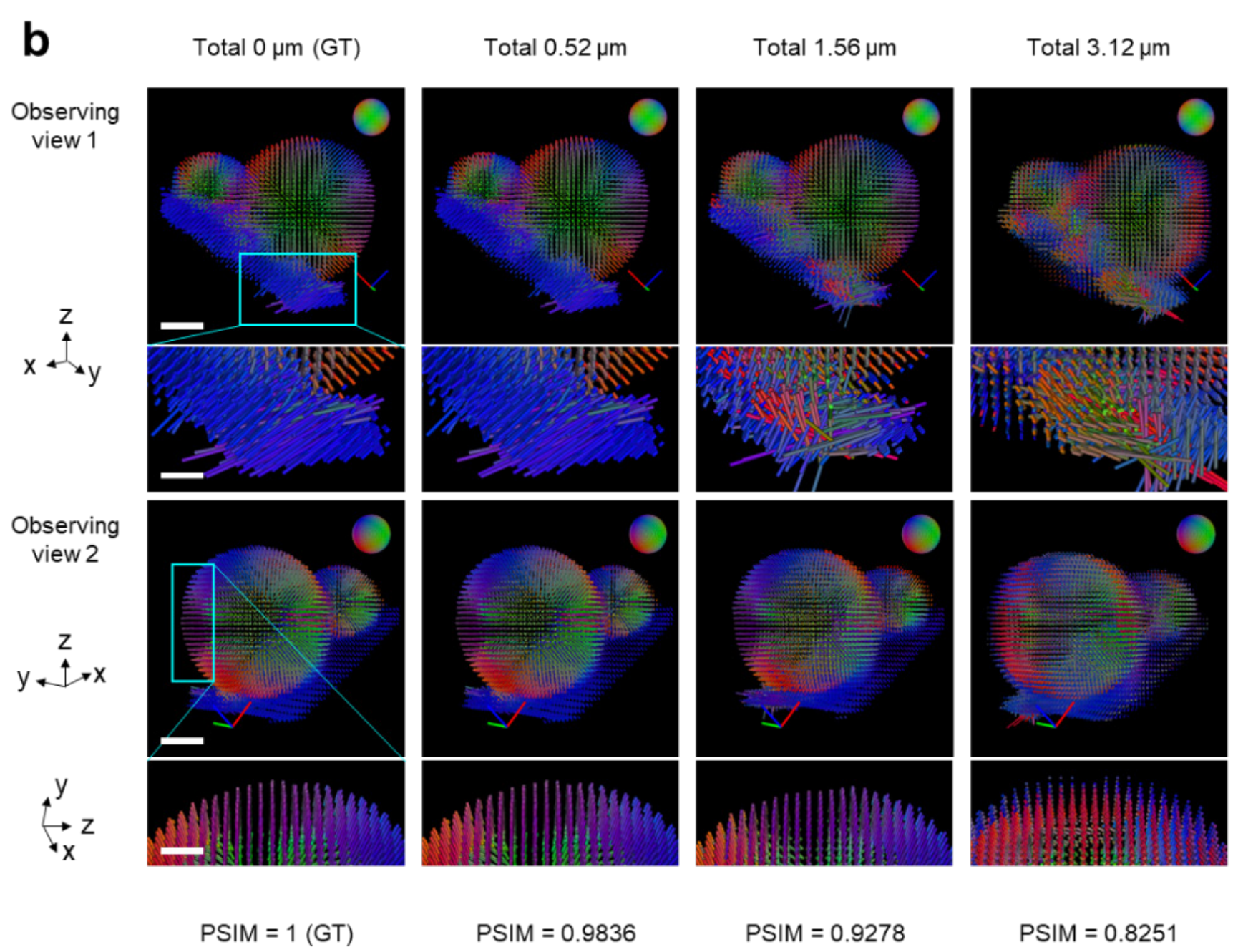

Revised **Supplementary Fig. 24**.

15. The legend of Fig. 1e should explain the representation which uses lines and colors, to make it understandable to the reader in all remaining figures.

RE 27: Following the reviewer's suggestion, we add the explanation to the caption of **Fig. 1e** while also leaving the more detailed explanation in **Supplementary Fig. 1**.
"**e)** ... Peak orientation maps with pseudo-color are demonstrated, in which the oriented cylinders marked by different colors indicate the most probable direction (ODF has the largest value in this direction) for fluorophores, and the length of cylinders indicates the relative degree of polarization (see also **Supplementary Fig. 1** for details)."

16. Some quantities are not defined, which makes it confusing to understand the equations which are manipulated in this work (e.g. in Eq. (37) does not define what is mu, V, U.. we can guess their meaning but it would be nice to state this in a rigorous way).

RE 28: Thanks for the careful reading and reminding us of these omissions. We now have gone through the equations and clarified the definitions.

For Eq. (11) in Line 554 on Page 14:
"where $|\boldsymbol{\tau}|$ and ϕ_τ denote the magnitude and angle of $\boldsymbol{\tau}$ in polar coordinates."

For Eq. (13) in Line 563 on Page 14:
"where w_0 is the beam waist radius and $\hat{\mathbf{z}}$ denotes the unit vector along z axis."

For Eq. (14) in Line 568 on Page 14:
"where $\hat{\mathbf{x}}$ denotes the unit vector along x axis..."

For Eq. (37) in Lines 674-675 on Page 18:
"where μ_k, $V_{k,\hat{\mathbf{p}}}$ and $U_{k,lm}$ are the k-th singular value, right singular vector and left singular vector of $\mathrm{H}_{lm,\hat{\mathbf{p}}}$, respectively. R is the rank of matrix $\mathrm{H}_{lm,\hat{\mathbf{p}}}$."
For Eq. (38) in Line 678 on Page 18:
"where η is a regularization parameter working for suppressing noise."

Reviewer #2 (Remarks on code availability):
I have not runned the code but I have gone through the information and parts of the code lines: it is very well documented.

RE 29: We thank the reviewer for carefully checking the code and document. We have also asked our collaborators to run and test through the code.

The authors extend their method for simultaneous 3D spatial and orientational fluorescence microscopy (reported previously in Chandler et al., PNAS 2025) by introducing an efficient generalized Richardson-Lucy (eGRL) deconvolution method to greatly improve accuracy in object estimation over the previous SVD method without much penalty in computing time and memory. The significance of this result is made apparent by the plethora of simulated and experimental demonstrations provided. It will pave the way for wider adoption of their technique. Moreover, the generalization of the RL algorithm to dimensions >3 seems significant to me from an image processing perspective.

In short, the manuscript is a tour de force and it deserve to be accepted with minimal revisions. I have only very minor questions and comments:

- Can the authors clarify their enumeration of "six dimensions" referred to in the title and abstract? 3 spatial + 2 orientation = 5 dimensions. In other works on orientational imaging they also quantify the "wobble angle", which I understand to be the 6th dimension in those papers. Of course, in the current work they go far beyond wobble angle to characterize the ODF in terms of (I think) 1+5+9 = 15 spherical harmonic coefficients.
- Line 151: "we noticed" should probably be "we note".
- Line 153: "The microscopy" should be "The microscope".
- Line 445: "orientational selectivity consistent" should probably be "consistent orientational selectivity".
- Can the authors clarify whether they are coining the term "fluorescent lake"? I haven't heard that before. I think they just mean it to refer to a featureless sample.
- Line 755: Generalized fractional anisotropy is a biological metric"—what is meant by "biological metric"? I think GFA comes from diffusion MRI so maybe it would be more precise to say this along with a citation.
- Line 785: "well-designed" should probably be "well-defined".
- In Supplementary Fig. 2 caption they should add a statement on the SSIM that appears in the figure.
- In Supplementary Fig. 4d the OSIM seems to asymptote to something below 1 (around 0.8). Why?
- In Supplementary Fig. 12a are the labels for "c" and "d" swapped?
